# Tunneling Splittings in the Water Hexamer Prisms Composed of Stacked Water Trimers

**DOI:** 10.1021/acs.jpca.5c06786

**Published:** 2025-12-11

**Authors:** Nina Tokić, Marko T. Cvitaš

**Affiliations:** Department of Physics, Faculty of Science, University of Zagreb, 10000 Zagreb, Croatia

## Abstract

We apply the modified WKB method to calculate tunneling splitting (TS) patterns in two hydrogen-bond isomers of the water hexamer, formed by the association of two water trimer rings stacked on top of each other with the same, clockwise–clockwise (CWCW), and the opposite, clockwise–counterclockwise (CWCCW), directionality of the in-ring hydrogen bonds. The TS patterns are composed of 78 (CWCW) and 90 (CWCCW) energy levels, which are interpreted in terms of the cluster rearrangements of flips, bifurcations and monomer rotations between equivalent symmetry-related minima of the cluster. In the vibrational ground state and the low-lying vibrationally excited states, we observe that two different monomer rotations, that both break two hydrogen bonds, explain the main features of the observable TS pattern in the CWCW form. In the CWCCW form, the dominant mechanism is a double flip, while several other mechanisms compete resulting in qualitatively different TS patterns in different vibrational states. The overall width of the TS pattern is larger in the more stable CWCCW form. Mild enhancements of the TS widths are observed in the fifth and sixth excited mode (numbered by increasing energy) in CWCW (19×) and CWCCW (4.8×) isomeric forms, respectively. We demonstrate that the present approach can be used to assign and interpret vibrational tunneling spectra of water clusters in terms of its distinct rearrangement mechaninsms when they compete for dominance in different low-lying vibrational states.

## Graphical abstract

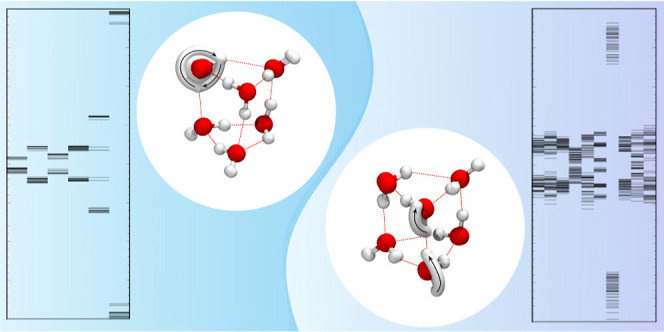

## 1 Introduction

Tunneling splittings (TSs) in water clusters have extensively been studied over the past few decades in experiments^1–5^ and using theory.^6–12^ The splittings of vibrational energy levels arise as a consequence of a tunneling interaction between the vibrational states localized in equivalent symmetry-related wells associated with different permutational isomers of the cluster.^6^ The interaction of the states belonging to different wells takes place in the overlap region, inside the barrier that separates them. The resulting energy splittings typically vary in size over many orders of magnitude^3,6,13–15^ and are particularly sensitive to the barrier height and shape. The structure of the TS pattern thus encodes the connectivity of different isomeric forms of the cluster via feasible tunneling rearrangements, as well as the water interactions at geometries that are distant from the stable minima, whose properties have been the focus of most studies.^16–20^ Development of accurate models of water interactions^21–28^ has driven the computational studies that calculate, assign and interpret vibrational spectra obtained using high-resolution spectroscopies. This has motivated the efforts to advance theoretical methods capable of treating high-dimensional systems,^9,29–32^ such as water clusters, and to compute the spectra that test the underlying water interaction models.

Early studies of TSs focused on water dimer.^6,7^ The vibrational spectrum was solved using numerically exact variational methods^33,34^ with a satisfactory agreement with experimental results. Water trimer has been solved using reduced-dimensional models; early attempts used three dimensions,^35,36^ while only recently a good agreement with experiment was obtained using 9 ^37^ and 12 ^38^ dimensions (with rigid monomers), in studies that also obtain the fine splittings due to bifurcations, the rearrangements that break hydrogen bonds.

The calculation of TSs requires electronic energies calculated using a high level of electronic structure theory (e.g., based on coupled-cluster methods that include up to triple electron excitations and large basis sets^27^), which is computationally feasible, for a large number of nuclear geometries and using well established methods, only for small clusters, consisting of up to three or four water molecules. Thus, larger clusters can be treated *ab initio* due to the development of electronic potentials that employ many-body expansions. Several state-of-the-art potential energy surfaces (PESs) based on that methodology, where few-body terms (up to the four-body) were fitted to permutationally invariant polynomials, have been developed under names CC-pol,^21^ WHBB,^22^ ,^23^ MB-pol^24–26,28^ and q-AQUA.^27^ Due to an unfavorable scaling of variational methods, TSs in larger clusters have been approached via diffusion Monte Carlo,^29^ path integral molecular dynamics^30^ (PIMD) or approximate semiclassical methods.^9,39–41^ These methods also rely on the tunneling matrix (TM) model^42^ in which the TS pattern of a multiwell system is obtained by calculating tunneling interactions between the pairs of states belonging to different wells. The calculations are thus separately conducted for each pair at a time in the limited regions of configuration space. TM model was used to calculate the ground-state (GS) TS in water dimer, trimer and the hexamer prism using numerically exact PIMD calculations.^10^ Approximate semiclassical instanton method^43^ is suited for calculations in high-dimensional systems, as it is based on an optimization of a tunneling path (in full dimensionality), rather than the sampling of a high-dimensional PES. It has been employed to find the relevant mechanisms and the GS TS patterns in the water dimer, trimer,^9^ pentamer^44^ and several structural isomers of the water hexamer and decamer,^45^ in a qualitative agreement with experiments. In an exemplary study, the GS TS pattern of the lowest-energy structural isomer of the water hexamer prism,^4^ named PR1 and shown in Figure 1, was determined experimentally as a doublet of triplets. A double hydrogen bond breaking mechanism, unlike any previously reported in experiments, was found to be responsible for the fine triplet splitting, using instanton theory.

**Figure 1:**
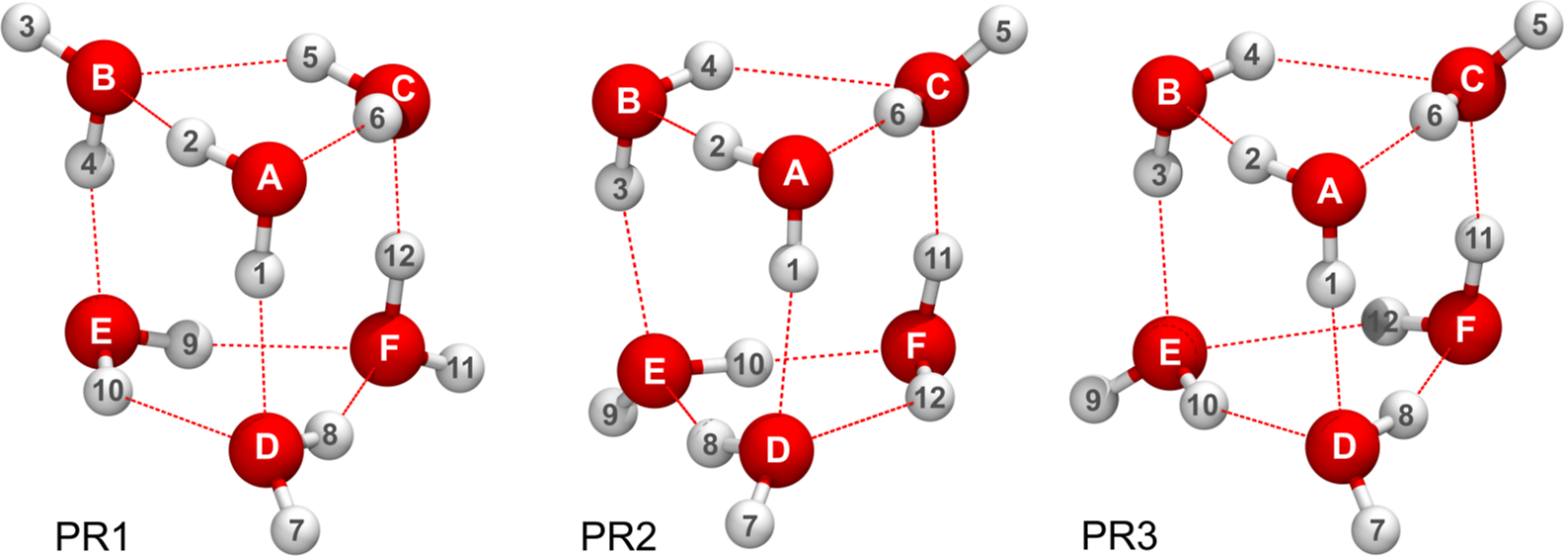
Minimum-energy geometry of the water hexamer prism isomers named PR1, PR2 and PR3, labeled in their reference versions.

The TS patterns in vibrationally excited states have also been investigated in several recent experiments. It was found that the excitation of a librational mode in water trimer,^3,13^ pentamer^14^ and the hexamer prism^46^ and cage^15^ results in 400–4000 times larger splittings relative to the GS TS. We recently used a modified WKB (M-WKB) approach,^41^ which gives identical results to instanton theory in the GS,^40^ to calculate the TS patterns in low-lying vibrationally excited states of water trimer^47^ and in the lowest-energy hexamer prism isomer^48^ PR1 in full dimensionality. M-WKB method fails to converge for high-frequency fundamentals. Because the observation of TSs in the excited low-frequency modes is challenging,^13^ it is not currently possible to compare the results for the excited states with experiment. This difficulty arises primarily from the limited line width and frequency stability of the available laser sources in the low THz region,^49^ combined with the small sizes of the splittings.

Reference 4 also found two other hydrogen bond network isomers of the water hexamer in their structural studies, PR2 and PR3 shown in Figure 1, with approximately the same hydrogen bond framework and at slightly higher energies. PR1–PR3 differ only in the orientation of the monomers and are difficult to distinguish by their rotational constants, primarily determined by their oxygen atom framework. It is however plausible that PR2 and PR3 can form in molecular beams since they can be formed by the association of two water trimer rings stacked on top of each other with the same directionality of hydrogen bonds in a clockwise–clockwise (CWCW) fashion in PR2, or with opposite directionality in a clockwise–counter clockwise fashion (CWCCW) in PR3. However, only the TS pattern of PR1 was detected in experiment.^4^ Reference 45 went further and proposed mechanisms that contribute to the formation of the GS TS pattern using instanton theory. It concluded that the TS pattern of PR2 is going to form a doublet, arising from the rotation of one of its monomers, while the TS pattern of PR3 is going to be complex, as several mechanisms that connect 384 permutational isomers of PR3 have competing TM element sizes.

The lowest-energy isomers of the water decamer are composed of two stacked water pentamer rings in CWCW and CWCCW forms that are ≈45 cm^–1^ apart in energy.^5,16^ These pentagonal prisms are analogous structures to the trigonal prisms of the hexamer isomers PR2 and PR3. GS TS patterns of both decamer isomers were calculated in ref 45. They both form a sextet, whereby the CWCCW form has twice the width of the CWCW form, in agreement with experiment.^5^ Monomer rotations, there, do not play part in the formation of the TS pattern. Their TS patterns are qualitatively similar to that of the water pentamer formed by “free” OH bond flips^44^ (as in the water trimer). There were also experimental attempts to measure the GS TS patterns in the lowest structural isomers of the water heptamer,^50^ octamer^51^ and nonamer^5^ without a successful detection.

The subject of this paper is the study of the TS patterns in the hexamer prisms PR2 and PR3. We apply the M-WKB method^40,41^ with MB-pol PES^24–26^ to determine the TM elements for the GS and several lowest-frequency fundamental excitations, and use group-theoretic analysis to determine the associated TS patterns. The objective is to investigate how the TS patterns and the relevance of the underlying mechanisms change upon vibrational excitation of the cluster and also across different hexamer isomers, in a follow-up to our recent study of the water hexamer prism PR1.^48^

The methodology that we use to determine the TS patterns is described in Section 2, followed by the presentation of results in Section 3. TS patterns for the hexamer prism PR2 are given in Section 3.1 and that for the PR3 is Section 3.2. Final conclusions are drawn in Section 4.

## 2 Methodology

In systems with multiple equivalent symmetry-related minima separated by large potential barriers, such as the water hexamers, degenerate localized single-well states interact via tunneling. These interactions cause energy shifts in the vibrational spectra and eigenstates delocalize over the system in symmetry-adapted linear combinations of the localized single-well states. We use the TM model^42^ in which the molecular Hamiltonian is represented in the basis of localized single-well states ϕ^(*i*)^. Rows and columns of the matrix are labeled by the equivalent minima *i*. A separate TM is constructed for each single-well vibrational state of interest and the interactions between different vibrational manifolds are neglected. Diagonal local energies are degenerate, set to zero, and taken as a reference energy. Off-diagonal tunneling interaction between a pair of local single-well states, belonging to minima *i* and *j*, is evaluated using Herring formula^52,53^

1 $$h_{ij}=\frac{1}{2}\int \left(\phi^{\left(j\right)}\left(n\nabla \right)\phi^{\left(i\right)}-\phi^{\left(i\right)}\left(n\nabla \right)\phi^{\left(j\right)}\right)\;d\Sigma$$

where Σ is the dividing surface, with unit normal ***n***, placed inside the barrier which separates the two wells. Equation 1 assumes the use of Cartesian mass-scaled coordinates and atomic units (ℏ = 1).

Single-well localized wave functions ϕ^(*i*/*j*)^ are obtained using our recently developed M-WKB method.^40,41^ The method is based on the multidimensional instanton theory by Mil’nikov and Nakamura^54^ and its extension to the vibrationally excited states.^55^ References 40 and 41 generalize the method to systems with multiple minima, such as the water hexamers, which exhibit asymmetry of the potential along the tunneling path, and treat the longitudinal and transverse vibrations (relative to the tunneling path) on equal footing. Further details of the methods are given in the above references, as well as ref 56. We only give a brief summary below.

The wave functions ϕ^(*i*)^ and ϕ^(*j*)^ are constructed along the minimum action path (MAP) that connects minima *i* and *j*. The MAP is a classical trajectory in the *f*-dimensional mass-scaled Cartesian coordinate space of molecular geometries ***x*** (*S*) (*f* = 54 for water hexamer) on the inverted potential, −*V*, which starts off at minimum *i* with zero kinetic energy and leads to minimum *j*. It is parametrized by the arc-length distance *S* from one minimum at *S* = 0 to the other minimum at *S* = *S*_max_. The Euclidean action is given by

2 $$A=\int_{0}^{S_{\max}}p\left(S^{\prime }\right)\;dS^{\prime }$$

In eq 2, ***p*** (*S*) is the magnitude of the *f*-dimensional momentum at ***x*** (*S*)­

3 $$p\left(S\right)=\frac{dx\left(S\right)}{d\tau }=\frac{dx\left(S\right)}{dS}\frac{dS}{d\tau }=t\left(S\right)p\left(S\right)$$

In eq 3, τ is the elapsed time and ***t*** is tangent to the trajectory (or MAP) at *S*. Potential *V*(*S*) at ***x*** (*S*) is defined with respect to the minimum, so from the conservation of energy, we have

4 $$p\left(S\right)=\sqrt{2V\left(S\right)}$$

The wave function for the GS (ν = 0) and the first excited state (ν = 1) in the normal mode ***U***_e_ with frequency ω_e_ at any point ***x*** is taken in the following Gaussian form

5 $$\begin{array}{ll} \phi_{\nu }\left(x\right) & =\sqrt[{4}]{\frac{\det A_{0}}{\pi^{f}}}\sqrt{{\left(2\omega_{e}\right)}^{\nu }}{\left(F\left(S\right)+U^{T}\left(S\right)\Delta x\right)}^{\nu }\\ & \times e^{\frac{-1}{2}\int_{0}^{S}\frac{Tr\left(A\left(S^{\prime }\right)-A_{0}\right)}{p\left(S^{\prime }\right)}\;dS^{\prime }}e^{-\int_{0}^{S}p\left(S^{\prime }\right)\;dS^{\prime }-p^{T}\Delta x-\frac{1}{2}\Delta x^{T}A\left(S\right)\Delta x} \end{array}$$

where Δ ***x*** = ***x*** – ***x*** (*S*) is a displacement from the reference point on the path ***x*** (*S*) and we drop the indices (*i*/*j*) that label minima. The parameters *F*(*S*), ***U*** (*S*) and ***A*** (*S*) along the MAP are determined by the following equations

6 $$p\left(S\right)\frac{d}{dS}A\left(S\right)=H\left(S\right)-A^{2}\left(S\right)$$

7 $$p\left(S\right)\frac{d}{dS}U\left(S\right)=\omega_{e}U\left(S\right)-A\left(S\right)U\left(S\right)$$

With initial conditions ***U*** (0) = ***U***_e_ and $A\left(0\right)=\sqrt{H\left(0\right)}$ , where ***H*** (*S*) is Hessian of potential at ***x*** (*S*), and *F*(*S*) = ***U*** ^T^(*S*) ***p*** (*S*)/ω_e_. For a general ***x***, the reference point ***x*** (*S*) in eq 5 is the closest point to it on the MAP. The first two factors on the r.h.s. represent the normalization constant, tuned to match the harmonic-oscillator state at minimum. The next term is the prefactor for the first excited state, where *F*(*S*) quantifies the shift of the nodal plane away from the MAP and ***U*** (*S*) traces the direction of the nodal plane along the MAP. The exponential terms in eq 5 describe the change of the amplitude due to the effect of the change of zero-point energies of the local normal modes along the MAP.

TM element *ij* is evaluated by inserting the wave functions ϕ^(*i*)^ and ϕ^(*j*)^ of the form given by eq 5 in Herring formula (eq 1). Dividing surface Σ is set to be the plane orthogonal to the MAP at the maximum of the potential along the MAP, at *S* = *S*_Σ_. Due to the Gaussian shape of the wave functions, the product in Herring formula can be integrated over the (*f* – 1)-dimensional Σ analytically. For the excited states, mixed terms involving *F* ^(*i*/*j*)^ ***U*** ^(*j*/*i*)^ do not contribute as they are odd over the domain of integration. Pure *F*-terms give longitudinal contribution, while *U*-terms give contribution of transverse modes. The theory works in full dimensionality and in Cartesian coordinates. Use of Hessians along the MAP assumes that the electronic potential is quadratic in directions perpendicular to the MAP. The effect of overall rotations is assumed to be decoupled and is neglected.

Numerical evaluation of a TM element *ij* starts by locating the MAP that connects minima *i* and *j*. The initial path is discretized into *N* equally spaced molecular geometries and determined iteratively in a gradient-based search using string method.^57,58^ Convergence criterion is set to 10^–6^ a.u. for the largest magnitude of the perpendicular-to-path *Nf*-dimensional action gradient at a discretization point along the string. The MAP is converged by progressively increasing the number of discretization points at subsequent optimizations. Potential *V*(*S*) and Hessians ***H*** (*S*) are then evaluated at the discretization points and used to construct the cubic spline interpolants at MAP. These are needed to solve eq 6 for ***A*** ^(*i*/*j*)^(*S*) at both sides of the dividing plane. Next, interpolants for ***A*** ^(*i*/*j*)^(*S*) are constructed and used to solve eq 7 for each excited mode ***U***_e_ of interest. Due to ***p*** (0) = 0, there is a singularity at *S* = 0 in eqs 6 and 7 and the solution at a small *S* = ε is obtained using linearization as in ref 41. From there, eqs 6 and 7 are solved using adaptive step Runge–Kutta. TM elements can then be determined by evaluating the Gaussian integrals in Herring formula. We varied the number of discretization points in the interval *N* = 300–1200 and ε in the interval from 0.1 *m*_e_^1/2^ *a*_0_ to 10 *m*_e_^1/2^ *a*_0_ in our calculations below to check the convergence.

This process is repeated for all pairs of minima to determine the non-negligible TM elements. Each pair of equivalent minima is connected by a permutation-inversion symmetry element. The symmetry elements associated with nonzero TM elements are used to generate the molecular symmetry group of the molecule. Each symmetry element of the group corresponds to an equivalent minimum of the molecule. Rows and columns of TM are then labeled by the symmetry elements of the group. For each vibrational state of interest, TM elements are inserted into appropriate positions in the TM and its eigenvalues then yield the TS spectrum. The associated eigenvectors of TM determine the state symmetries.

## 3 Tunneling Splittings in the Hexamer Prisms PR2 and PR3

The lowest-energy isomer of the water hexamer is the hexamer prism PR1, shown in Figure 1 in its equilibrium geometry. In the two bases of the prism, water molecules C and E, labeled by their oxygen atoms, act as double donors to the in-base hydrogen bonds. This makes them different to the prisms PR2 and PR3 shown in Figure 1, where each water molecule acts as a single donor and acceptor in the in-base bonds. Each base has a qualitatively similar structure to the most stable ring form of the water trimer. The two trimer rings can be associated on top of each other with the same directionality of the in-ring OH bonds in PR2 (see Figure 1), or the clockwise–clockwise (CWCW) structure, or with the opposite directionality in PR3, or the clockwise–counterclockwise (CWCCW) structure. The potential energies of PR2 and PR3 at minimum are 481.7 cm^–1^ and 451.7 cm^–1^ higher than the minimum of PR1 and only 30 cm^–1^ apart. There are possibly many stable structures of the hexamer which lie at energies in between, including the hexamer cage (89.1 cm^–1^ above PR1) and the book (369 cm^–1^). If harmonic zero-point energy is included, the GSs of PR2 and PR3 are 420.3 cm^–1^ and 381.3 cm^–1^ above the GS energy of PR1, respectively.

TS pattern of water trimer has been determined in experiment and computed in 9- and 12-dimensional variational calculations.^37,38^ It has also qualitatively been described using instanton theory via the rearrangement dynamics of flips and bifurcations.^9^ In the trimer ring, each water molecule has one hydrogen upholding the in-ring hydrogen bond and the other hydrogen pointing either above or below the ring plane. Flip is the motion in which a water molecule rotates about the in-ring hydrogen bond, whereby “free” hydrogen (not involved in hydrogen bonding) moves from one to the other side of the ring plane. The torsional motion of the flip does not break any bonds. Bifurcations, on the other hand, are mechanisms that involve the rotation of both hydrogen atoms about the axis perpendicular to the water molecule and passing through its oxygen atom. The in-bond hydrogen moves outside the ring plane, breaking the hydrogen bond, and is replaced by the other hydrogen atom that moves into its place in the ring plane from the opposite side of the plane. It was found that bifurcations in water trimer can be accompanied by one or more simulataneous flips.^9^ Flips in water trimer are “free” (nontunneling) motions because the zero-point energy is higher than the potential barrier along the rearrangement path. This compromises the accuracy of the instanton calculations and limits the agreement with the formally exact variational methods and PIMD.^10,59^ Similar monomer dynamics is present in other water clusters, in particular in the water pentamer^44^ and hexamer prism PR1.^4^

In the hexamer prism PR1, the doublet splitting in experimental spectra has been explained by a simultaneous trimer-like flip of water molecules A and D that lie on two different bases and along the same side of the prism.^4^ Unlike the water trimer, the flip motion here does break and reform a hydrogen bond. A simultaneous bifurcation of water molecule A and a flip of water molecule D (or vice versa), denoted ÃD/AD̃, where ∼ above the monomer symbol denotes a bifurcation, was found to be responsible for the fine splitting of each doublet level into a triplet. This mechanism breaks two hydrogen bonds and has been observed in the spectrum with peaks separated by 4.9 × 10^–6^ cm^–1^. Instanton theory gives ≈30% larger separations. All tunneling pathways in the water hexamer involve large potential barriers and comparison with the exact PIMD results for the GS^10^ have shown that instanton theory yields quantitatively accurate predictions (within a factor of 2). In our previous study, we estimated, using M-WKB method, that the rotations of double in-plane donor water monomers C and E around their *C*_2_ axis, the “rot C” and “rot E” mechanisms, produce a further triplet splitting of each triplet state.^48^ Using dipole selection rules, this does not qualitatively change the observed spectrum, but the experimental evidence for these mechanisms is missing. We also calculated the TS patterns in 13 low-lying vibrationally excited states.^48^ A previous M-WKB study of TSs in vinyl radical^56^ shows that the theory is able to reproduce large enhancements of TSs of more than 1000×, whenever they occur. We found that the TM elements in PR1 can be ≈50× reduced and ≈10× enhanced relative to the GS, depending on which mode of vibration is excited. In some vibrational states, bifurcations and rotations are predicted to outweigh the effect of the flip mechanism in shaping of the TS pattern.

### 3.1 Tunneling Splittings in the Hexamer Prism PR2 (CWCW)

The hexamer prism PR2, labeled in its reference version, is shown in Figure 1. The mechanisms of flips, bifurcations and rotations of single water monomers have been identified in ref 45. We performed an independent search for MAPs that connect pairs of minima by processes analogous to those in water trimer (or in hexamer PR1). A pair of minima is linked by a string of discretization points, the action is iteratively minimized and TM elements determined using M-WKB, as described in Section 2. In Figure 2a–f, we show the superposed snapshots of molecular geometries along the MAPs for six rearrangement mechanisms that result in the largest TM elements *h*.

**Figure 2:**
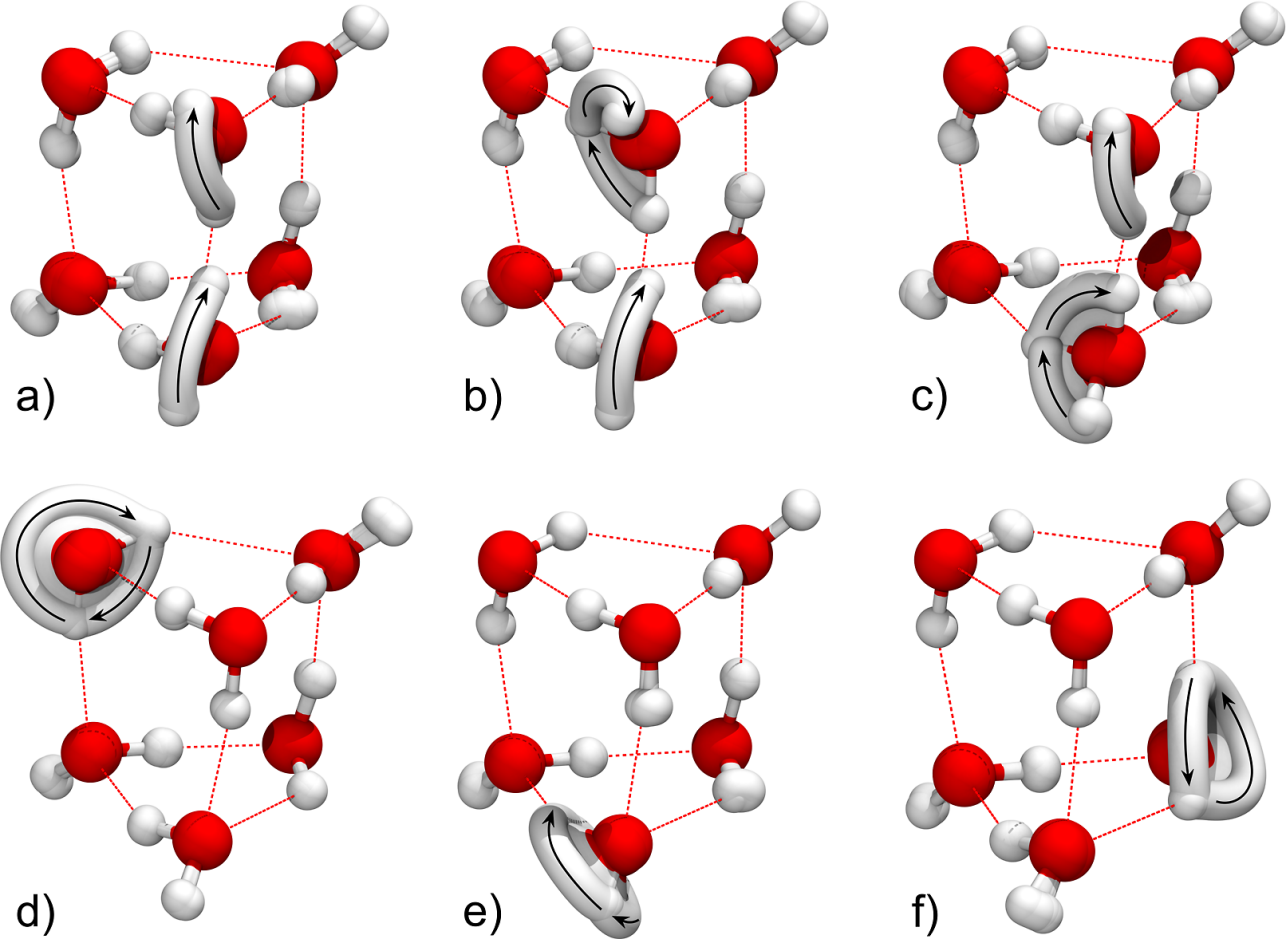
Minimum action paths of six rearrangements in the water hexamer prism PR2 that are responsible for the formation of the tunneling splitting pattern: (a) double flip AD/BE, (b) simultaneous bifurcation and a flip ÃD/BẼ, (c) AD̃/B̃E, (d) monomer rotation rot B, (e) rot D and (f) rot F.

A double-flip mechanism is shown in Figure 2a and it involves a simultaneous flip of monomers A and D, denoted AD, whereby one hydrogen bond breaks. The rearrangement links the reference version in Figure 1 to the permutational isomer obtained by the symmetry element (AFBDCE) (1 11 3 7 5 9)(2 12 4 8 6 10)*, where * denotes inversion. The cycle length is 6 which means that 6 subsequent AD processes lead back to the reference version in a sequence that links 6 different symmetry-related minima. The inverse symmetry element is associated with the mechanism BE, a double flip of monomers B and E, which therefore has the same TM element size as the mechanism AD, and the subsequent processes link the same 6 minima in the opposite order. The situation is analogous to the flip-induced TS pattern in water trimer, where 6 subsequent flips lead back to original version. Thereby a chosen monomer makes two circles around the ring. After the first full circle the structure is inverted. If only the AD/BE mechanism was present in the hexamer prism PR2, it would exhibit a qualitatively similar TS pattern to water trimer; a quartet with two central states doubly degenerate and a significantly reduced width compared to water trimer, due to a high barrier involved in the breaking of a hydrogen bond.

Mechanism shown in Figure 2b consists of bifurcation of monomer A accompanied by a flip of monomer D, ÃD. The rearrangement links the reference version in Figure 1 to the permutational isomer obtained by the symmetry operation (AFBDCE)(1 11 3 7 5 9 2 12 4 8 6 10)*. The cycle length is 12 which means that 12 subsequent ÃD processes lead back to the reference version passing through 12 different permutational isomers. The inverse symmetry element is associated with mechanism BẼ, which therefore has the same TM element size. Monomer A thereby passes through all the vertices of the prism twice. After one full cycle over the vertices, hydrogen atoms on all monomers switch places, i.e., this version is linked by the symmetry element (1 2)(3 4)(5 6)(7 8)(9 10)(11 12) to the reference in Figure 1. The TS pattern associated with the ÃD/BẼ mechanism alone is a septet with energy levels

$$2h_{ÃD}:\sqrt{3}h_{ÃD}:h_{ÃD}:0:-h_{ÃD}:-\sqrt{3}h_{ÃD}:-2h_{ÃD},$$

whereby the outer levels are nondegenerate and the inner levels are doubly degenerate.

Mechanism in Figure 2c is labeled AD̃ and its inverse is B̃E. Both involve a bifuraction (D̃ or B̃) accompanied by a simultaneous flip (A or E). AD̃ is associated with the symmetry element (AFBDCE)(1 11 3 8 6 10 2 12 4 7 5 9)* of cycle length 12, so it also links 12 different minima, as do ÃD/BẼ mechanisms, and, on its own, produces a septet of states. Two bifurcation mechanisms ÃD/BẼ and AD̃/B̃E, taken together, connect 48 permutational isomers and span a 48-element group.

Figure 2d–f show *C*_2_ rotations of monomers B, D and F, associated with (3 4), (7 8) and (11 12) transpositions. Below, they are referred to as “rot B”, “rot D” and “rot F”, respectively. They link pairs of minima and, on their own, each produce a doublet splitting.

Potentials along MAPs for the above mechanisms are shown in Figure 3. The barriers for AD/BE, ÃD/BẼ, AD̃/B̃E, rot B, rot D and rot F are, respectively, 2122 cm^–1^, 2188 cm^–1^, 1838 cm^–1^, 1627 cm^–1^, 2113 cm^–1^ and 1751 cm^–1^. The MAP lengths vary from 323.0 *m*_e_^1/2^ *a*_0_ for AD to 417.5 *m*_e_^1/2^ *a*_0_ for rot F. In comparison, rot C and rot E mechanisms in PR1 have similar barrier heights and widths to rotations in PR2, while AD and ÃD/AD̃ mechanisms have smaller and narrower barriers, and are dominant.^48^ Bifurcations in water trimer, which break one hydrogen bond, have barriers of ≈800 cm^–1^. Harmonic frequencies of the normal modes are listed in Supporting Information in Table S1. All vibrational states corresponding to a single excitation of one of the 30 intermonomer vibrational modes, with frequencies up to 943.1 cm^–1^, are tunneling states.

**Figure 3:**
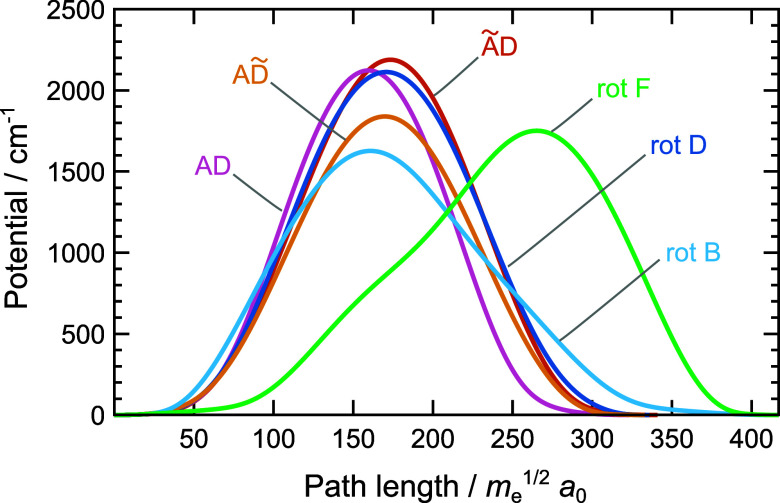
Potential energy curves along the minimum action paths for different rearrangement mechanisms in the water hexamer prism PR2, shown in Figure 2. See text for notation.

We calculated the TM elements using M-WKB in the GS and the fundamental excitations of the five lowest-frequency vibrational modes and list them in Table 1. They are shown graphically in Figure 4. Modes ν_1_–ν_5_, numbered in the order of increasing energy, lie in the range from 61.8 cm^–1^ to 92.1 cm^–1^. TM elements for the excited modes having higher frequencies do not converge for some of the dominant mechanisms. Reference 60 ascribes this to the failure of WKB approximation in the present form. The uncertainty in the size of the small projections of the normal modes on the MAP at ε due to finite precision, in combination with large imaginary times involved to reach the dividing plane, lead to a numerical instability^47^ due to exponential rise of *F*(*S*_Σ_) = *F*(ε) exp­(ω_e_τ). On the other hand, using larger ε values leads to inconsistent treatment of rotations.

**Table 1:** Tunneling Matrix Elements of the Water Hexamer Prism PR2 on MB-pol PES^24–26^ for Rearrangement Paths AD, ÃD, AD̃, Rot B, Rot D and Rot F in the Vibrational Ground State and the 5 Lowest-Frequency Excited Vibrational Modes in cm^–1^

mode	*h*_AD_/10^–7^	$h_{ÃD}/10^{-8}$	$h_{ÃD}/10^{-7}$
GS	–8.91	–5.81	–8.97
1	5.51	3.49	7.49
2	0.578	3.44	–2.67
3	2.94	–3.02	–8.71
4	–6.76	–3.21	–2.21
5	13.4	3.85	6.38

mode	*h*_rot B_/10^–5^	*h*_rot D_/10^–7^	*h*_rot F_/10^–6^
GS	–0.700	–8.11	–0.859
1	–1.96	–12.1	–1.31
2	–0.959	–9.18	–1.92
3	–1.91	–11.5	–0.888
4	–3.94	–19.9	–19.8
5	–18.8	–11.3	–6.32

**Figure 4:**
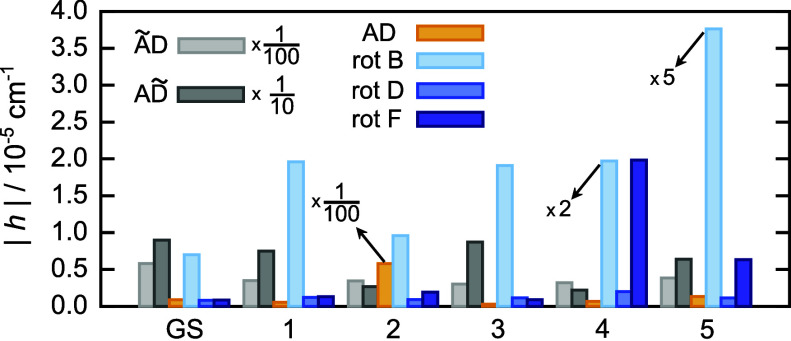
Tunneling matrix elements *h* of the water hexamer prism PR2 obtained using M-WKB on the MB-pol PES for six rearrangement paths, AD, ÃD, AD̃, rot B, rot D and rot F, shown in Figure 2 in the vibrational GS and the five lowest-frequency excited modes, ν_1_–ν_5_, in cm^–1^.

TM elements in the GS are in close agreement with ref 45 obtained using ring-polymer instanton theory. The TM elements for flips/bifurcations are within 11–13%, while rot B is 5.7% smaller and rot D and rot F are 30% larger here. We note that the TM elements for rotations have been multiplied by 2 in Table 1 to account for the two senses of rotation, as in ref 9 for the acceptor tunneling in water dimer. Both senses of rotation connect the same minima by paths passing through different geometries in the configuration space and contribute equally.

TSs in the excited states of water clusters, at the excitation frequencies in excess of 500 cm^–1^ exhibit large enhancements relative to the GS TS.^13–15,46^ Here, we observe that the TM elements for flips and bifurcations in the excited modes ν_1_–ν_5_ are reduced, apart from only the TM element for AD in ν_5_ (with a 50% increase relative to the GS), as seen in Table 1. The excitation of the longitudinal mode, the one with the largest projection on the MAP at minimum, usually results in a TM element of increased size, due to the *F* term in eq 5. In PR2, there is no clear longitudinal mode for any of the mechanisms and the path direction is spanned by the modes ν_1_–ν_5_ at minimum and no such increase is observed. TM elements for rotations, on the other hand, increase in size, relative to the GS, in each of the excited modes considered here. The most notable increases are for rot B in the excited mode ν_5_, by ≈27× and for rot F in the excited ν_4_, by ≈23×.

We also considered other mechanisms such as ÃD̃/B̃Ẽ, rot A, rot C and rot E. The double bifurcation mechanisms proceeds over the barrier of 3391 cm^–1^ and gives TM element *h* = 2.3 × 10^–12^ cm^–1^. Rot A, rot C and rot E have barriers along the MAP of 3071 cm^–1^, 3019 cm^–1^ and 3956 cm^–1^, with the GS TM elements of 1.0 × 10^–9^ cm^–1^, 1.0 × 10^–8^ cm^–1^ and 9.7 × 10^–11^ cm^–1^, respectively. They are thus considered unfeasible here and are neglected.

Inclusion of all TM elements considered above generates a group *G*_384_ of 384 elements. Monomer A can take 6 positions at the vertices of the prism and a transposition of hydrogens on each monomer generates additional 2^6^ versions; 6 × 2^6^ = 384. Starting from the reference version, reaching any of the 384 minima requires a maximum of 6 single-step processes. Only two mechanisms are required to span *G*_384_ group, AD and another flip/bifurcation mechanism, or one of three flip/bifurcation mechanisms combined with a rotation. The group *G*_384_ divides into 28 classes, defined in Supporting Information in Table S2. Using Burnside’s algorithm, we generated the character table of the group in Table S3 in Supporting Information and defined the labels of irreducible representations (irreps) of the group. Symmetries of tunneling states, Γ_tun_, are found by reducing the representation of the full basis set, composed of one localized single-well state per minimum.^12^ It has character 384 for identity and zero for all other symmetry elements. Reduction gives Γ_tun_ = *A*_1_^+^ ⊕ *A*_1_^–^ ⊕ *A*_2_^+^ ⊕ *A*_2_^–^ ⊕ *E*_1_^+^ ⊕ *E*_1_^–^ ⊕ *E*_2_^+^ ⊕ *E*_2_^–^ ⊕ 2*E*_3_ ⊕ 3*T*_1_^+^ ⊕ 3*T*_1_^–^ ⊕ 3*T*_2_^+^ ⊕ 3*T*_2_^–^ ⊕ 2*G*_1_ ⊕ 6*I*_1_ ⊕ 6*I*_2_ ⊕ 6*I*_3_ ⊕ 6*I*_4_ ⊕ 6*I*_5_ ⊕ 6*I*_6_ ⊕ 6*I*_7_ ⊕ 6*I*_8_ ⊕ 6*I*_9_. There are thus 78 distinct states in the splitting pattern and the degeneracy of states can be 1 (*A* states), 2 (*E*), 3 (*T*), 4 (*G*) or 6 (*I*). Total wave function is a product of rovibrational state and the nuclear spin state and must be antisymmetric under exchange of hydrogen nuclei and symmetric under exchange of oxygen nuclei. Therefore, it must be either of Γ^+^ = *A*_2_^+^ or of Γ^+^ = *A*_2_^–^ symmetry. Nuclear spin states span Γ_nuc_ = 130*A*_1_^+^ ⊕ 119*A*_1_^–^ ⊕ *A*_2_^+^ ⊕ 248*E*_2_^+^ ⊕ 232*E*_2_^–^ ⊕ 11*E*_3_ ⊕ 45*T*_1_^+^ ⊕ 36*T*_1_^–^ ⊕ 6*T*_2_^+^ ⊕ 3*T*_2_^–^ ⊕ 16*G*_1_ ⊕ 243*I*_1_ ⊕ 81*I*_2_ ⊕ 81*I*_3_ ⊕ 27*I*_4_ ⊕ 27*I*_5_ ⊕ 27*I*_6_ ⊕ 9*I*_7_ ⊕ 9*I*_8_ ⊕ 3*I*_9_. We note here that there are no states of either *E*_1_^+^ or *E*_1_^–^ symmetry. In the ground rotational state, vibrational tunneling states have the following statistical weights, Γ_vib_ = *A*_1_^+^ ⊕ *A*_1_^–^ ⊕ 249*A*_2_^+^ ⊕ 249*A*_2_^–^ ⊕ 480*E*_1_^+^ ⊕ 480*E*_1_^–^ ⊕ 22*E*_3_ ⊕ 9*T*_1_^+^ ⊕ 9*T*_1_^–^ ⊕ 81*T*_2_^+^ ⊕ 81*T*_2_^–^ ⊕ 32*G*_1_ ⊕ 6*I*_1_ ⊕ 18*I*_2_ ⊕ 18*I*_3_ ⊕ 54*I*_4_ ⊕ 54*I*_5_ ⊕ 54*I*_6_ ⊕ 162*I*_7_ ⊕ 162*I*_8_ ⊕ 486*I*_9_. Levels *E*_2_^+^ and *E*_2_^–^ are missing from the spectrum. Assuming that the dipole moment vector lies parallel to the prism *C*_3_ axis, the symmetry elements of classes 21–28, see Table S2 in Supporting Information, change the direction of the dipole moment. The dipole is therefore of *A*_1_^–^ symmetry.

We also worked out the state symmetries in the fully deuterated d12-prism PR2, although TSs in this system are inobservable and have not been computed here. Total internal wave function is then symmetric under exchange of hydrogens and oxygens and is thus of either Γ^+^ = *A*_1_^+^ or Γ^+^ = *A*_1_^–^ symmetry. In the ground rotational state, Γ_vib_ = 15576*A*_1_^+^ ⊕ 15576*A*_1_^–^ ⊕ 249*A*_2_^+^ ⊕ 249*A*_2_^–^ ⊕ 480*E*_1_^+^ ⊕ 480*E*_1_^–^ ⊕ 3912*E*_3_ ⊕ 31080*E*_2_^+^ ⊕ 31080*E*_2_^–^ ⊕ 11664*T*_1_^+^ ⊕ 11664*T*_1_^–^ ⊕ 2916*T*_2_^+^ ⊕ 2916*T*_2_^–^ ⊕ 7752*G*_1_ ⊕ 46656*I*_1_ ⊕ 23328*I*_2_ ⊕ 23328*I*_3_ ⊕ 11664*I*_4_ ⊕ 11664*I*_5_ ⊕ 11664*I*_6_ ⊕ 5832*I*_7_ ⊕ 5832*I*_8_ ⊕ 2916*I*_9_. All levels are populated in d12-prism PR2.

Degenerate states of permutational isomers form a basis in which we construct the TM matrix of dimension 384 × 384. Each row/column of the matrix has 9 nonzero TM elements, while 6 of them are distinct; AD/BE, ÃD/BẼ and AD̃/B̃E account for a pair of identical elements each, and rot B, rot D and rot F for one nonzero TM element each. Eigenvalues of TM for each vibrational state (GS and ν_1_–ν_5_) give us the tunneling spectrum, which is shown in Figure 5 in the right panel. Eigenvectors of TM determine the state symmetries. There are 78 nondegenerate states in each vibrational manifold. Their energies, relative to the degenerate localized vibrational state energies, and state symmetries are listed in Tables S4–S9 in Supporting Information (using rounded TM elements as shown in Table 1).

**Figure 5:**
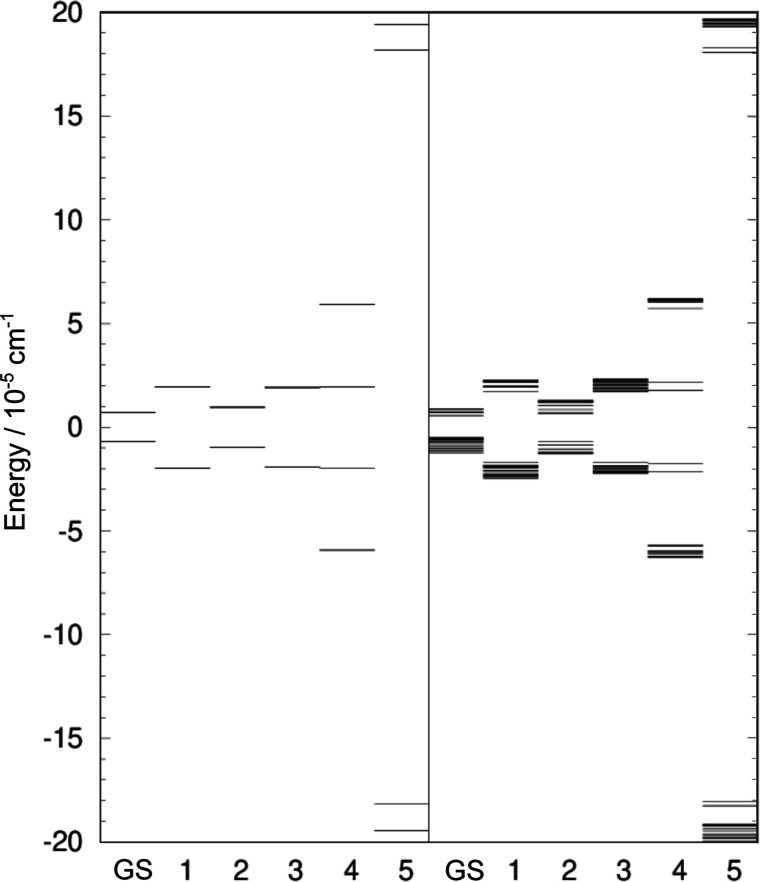
Tunneling splitting (TS) patterns of the water hexamer prism PR2 in the vibrational GS and the excited vibrational modes ν_1_–ν_5_. Left panel shows the TS pattern in which the contribution of the rot B mechanism is included in all states and, additionally, the rot F in the excited modes ν_5_ and ν_6_. Right panel shows the TS pattern which includes contributions of all six rearrangement mechanisms considered in the text and shown in Figure 2. See text for notation.

The TS pattern in Figure 5 (right) reveals a doublet structure in GS and the excited state ν_1_–ν_3_, where each branch is further split into a large number of states (39 in each branch). The TM element for the mechanism rot B is the largest for all vibrational manifolds shown in Figure 5 (right) and is responsible for the qualitative appearance of the TS pattern, as ref 45 suggests. The GS splitting results in the doublet width of 1.4 × 10^–5^ cm^–1^ (due to rot B), where the two branches have the widths of 7.4 × 10^–6^ cm^–1^ and 3.2 × 10^–6^ cm^–1^. The branch with the higher energy is narrower, as observed in the GS of water trimer^47^ and pentamer,^44^ because a number of competing mechanisms enter the energy expression with preferentially different signs resulting in cancellations. The mechanisms rot F, rot D, AD̃ and AD, in this order, contribute to the further and finer splittings. Overall width of the GS TS pattern is 2.1 × 10^–5^ cm^–1^ (see Table S4 in Supporting Information). The widths in the excited states ν_1_ and ν_3_ are ≈2× larger.

In the excited mode ν_4_ and ν_5_, TS pattern displays a qualitative change signifying that other mechanisms also play an important part in its formation. Inspection of Table 1 reveals that the simple structure of the TS pattern in the excited mode ν_4_ is a doublet of doublets. Mechanism rot B produces a doublet of states separated by 2|*h*_rot B_| = 7.9 × 10^–5^ cm^–1^, and each doublet branch is further split by the mechanism rot F into the doublets of width 2|*h*_rot F_| = 4.0 × 10^–5^ cm^–1^. TM elements for rot B increases by a factor of 5.6, relative to the GS, while the TM element for rot F increases 23× and becomes comparable in size. Further fine splittings in the TS pattern in the excited mode ν_4_, in Figure 5 (right), reveal the signature of the mechanism rot D. Similarly, the mechanisms rot B and rot F are responsible for the appearance of the quartet in the excited mode ν_5_. The overall width in this state is the largest at ≈4 × 10^–4^ cm^–1^. In the left panel of Figure 5, we show the TS pattern that is produced when we include the mechanism rot B in the GS and all excited states, and, on top of it, also the rot F mechanism in the excited states ν_4_ and ν_5_. In this way, the qualitative appearance of the full TS pattern on the right panel is reproduced.

In conclusion, as first proposed by ref 45, the TS pattern in the hexamer prism PR2 (CWCW) is primarily determined by rotation of monomer B, shown in Figure 2d. Contribution of the mechanism rot F is expected in the excited modes ν_4_ and ν_5_, while the largest enhancement of the TS width, relative to the GS, is anticipated in the excited mode ν_5_. Monomer rotations which break two hydrogen bonds are the dominant tunneling pathways in PR2. Similar mechanisms have never been observed in experiment and it is uncertain how well the PES is described along these tunneling pathways. A similar motion is the acceptor tunneling in water dimer, which is an overbarrier process and does not break any bonds.

We compare the GS TM elements obtained above using MB-pol^24–26,28^ PES with those obtained using WHBB^22,23^ PES. For this purpose, we reoptimized the MAPs for each of the mechanisms studied above using WHBB potential and list the GS TM elements for both potentials in Table S22 in Supporting Information. We note that the WHBB TM elements do differ from MB-pol values, but remain within 55% of the corresponding MB-pol TM elements for all the relevant mechanisms (in both, PR2 and PR3) with one exception. Specifically, the TM element for the mechanism AD̃ in PR2 is 2.2× larger. The difference can entirely be attributed to the difference in actions, as exp­(−Δ*A*) = 2.2 (actions in eq 2 are 21.85 ℏ (WHBB) and 22.65 ℏ (MB-pol)). While this means that the TS patterns might change in some regions, using a hypothetical exact potential, the relevant mechanisms and their significance, likely, will not. Similar relative differences in TM elements obtained using the two PESs were found in other water clusters.^45^

In comparison with the decamer prism composed of CWCW-stacked pentamer rings as bases, different mechanism dominate the splittings. In the decamer,^45^ the double flip mechanism dominates, with its TM element of 1 × 10^–5^ cm^–1^, resulting in the sextet that is 4 × 10^–5^ cm^–1^ wide. TM elements of bifurcation mechanisms are more than an order of magnitude smaller in the decamer, while that of the monomer rotations are of the order 10^–9^ cm^–1^ and negligible. Overall widths of the TS patterns are similar in the CWCW decamer and the CWCW hexamer (within a factor of 2). The strained structure of the hexamer changes the hydrogen bond strengths in such a way that, relative to the decamer, the effect of similar motions changes by orders of magnitude, resulting in a qualitatively different splitting pattern.

### 3.2 Tunneling Splittings in the Hexamer Prism PR3 (CWCCW)

The hexamer prism PR3, labeled in its reference version, is shown in Figure 1. The mechanisms of flip, bifurcations and monomer rotations have been identified in ref 45. In our independent search, we optimized the MAPs and show those that correspond to the largest TM elements in Figure 6a–g. Again, relevant motions are similar to those found in the hexamer prism PR1 ^4,48^ and the water trimer.^9^

**Figure 6:**
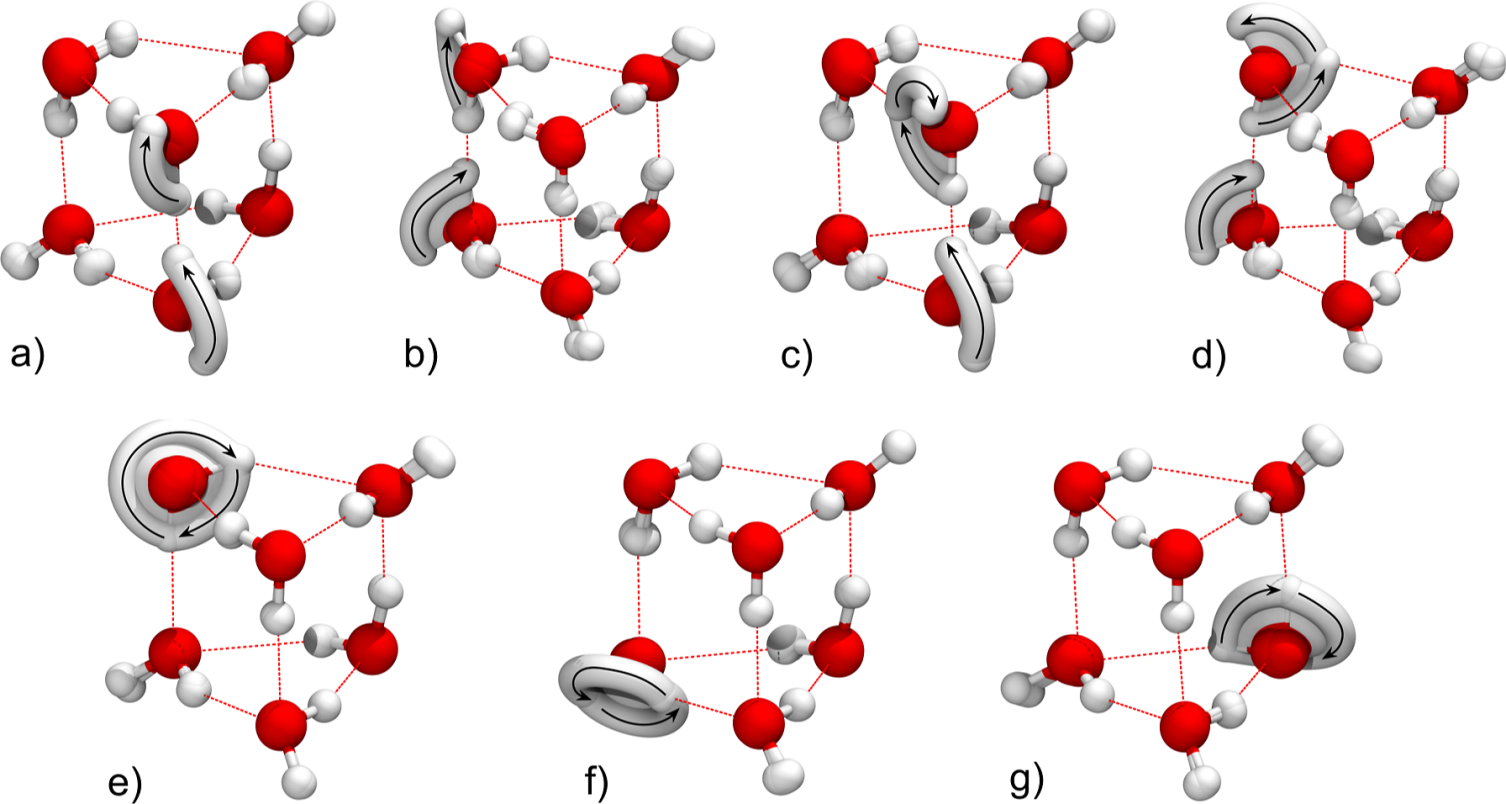
Minimum action paths of seven rearrangements in the water hexamer prism PR3 that are responsible for the formation of the tunneling splitting pattern: (a) double flip AD, (b) BE, (c) simultaneous bifurcation and a flip ÃD/AD̃, (d) B̃E/BẼ, (e) monomer rotation rot B, (f) rot E and (g) rot F.

A simultaneous double flip of monomers A and D, shown in Figure 6a breaks one hydrogen bond and links the reference version to the version obtained by the symmetry operation (AD)­(BF)­(CE)(1 7)(2 8)(3 11)(4 12)(5 9)(6 10). The operation is its own inverse and on its own makes a double-well system, same as the double-flip AD in the hexamer prism PR1.

Unlike the prism PR1, another double-flip mechanism is present here involving monomers B and E, shown in Figure 6b. The associated symmetry operation is (AF)­(BE)­(CD)(1 11)(2 12)(3 9)(4 10)(5 7)(6 8), again its own inverse and resulting in a double-well system. The two double-flips, taken together, give access to six different minima in which monomer A takes up positions at the 6 vertices of the prism. TS pattern is a quartet, as in water trimer, with the energy levels relative to the degenerate state given by

$$h_{AD}+h_{BE}:-\sqrt{{\left(h_{AD}-h_{BE}\right)}^{2}+h_{AD}h_{BE}}$$

$$:\sqrt{{\left(h_{AD}-h_{BE}\right)}^{2}+h_{AD}h_{BE}}:-\left(h_{AD}+h_{BE}\right)$$

where the two inner states are doubly degenerate.

Bifurcation of monomer A and a simultaneous flip of monomer D, ÃD, are shown in Figure 6c. The symmetry operation that links the two minima is (AD)­(BF)­CE)(1 7 2 8)(3 11)(4 12)(5 9)(6 10). Subsequent operations ÃD lead back to the original version after passing through 4 permutational isomers. The inverse operation is AD̃ and has the same TM element. On their own, the mechanisms ÃD/AD̃ split the degenerate vibrational state into a triplet, with relative energies $2h_{ÃD}:0:-2h_{ÃD}$ , where the mid level is doubly degenerate.

Simultaneous bifurcation of monomer B and a flip of monomer E, B̃E, is shown in Figure 6d. The associated symmetry operation is (AF)­(BE)­(CD)(1 11)(2 12)(3 9 4 10)(5 7)(6 8). It links 4 permutational isomers. The inverse operation is BẼ. As ÃD/AD̃, the mechanisms B̃E/BẼ alone induce a triplet splitting. The combination of a flip and bifurcation mechanism AD + ÃD/AD̃ or BE + B̃E/BẼ connect 8 permutational isomers, while AD + B̃E/BẼ or BE + ÃD/AD̃ connect 96 permutational isomers. A combination of two bifurcations B̃E/BẼ + ÃD/AD̃ connect 48 isomers. Any combination of 3 flip/bifurcation mechanisms connect 384 isomers.

Figure 6e–g show rotations of monomers B, E and F, rot B, rot E and rot F. The associated symmetry operations are (3 4), (9 10) and (11 12), respectively. Each mechanism on its own produces a doublet splitting. Any of the rotations combined with two flip/bifurcation mechanisms that involve monomers A, D, B and E connect 384 permutational isomers.

Potentials along MAPs for these mechanisms are shown in Figure 7. The barriers along MAPs for AD, BE, ÃD/AD̃, B̃E/BẼ, rot B, rot E and rot F are 1777 cm^–1^, 1738 cm^–1^, 1929 cm^–1^, 1300 cm^–1^, 1805 cm^–1^, 2148 cm^–1^ and 1842 cm^–1^, respectively. The MAP lengths vary from 281.8 *m*_e_^1/2^ *a*_0_ for the mechanism AD to 405.9 *m*_e_^1/2^ *a*_0_ for rot F. Rotations in PR3 have similar barriers heights to rotations in PR1 and PR2, while the barriers for flips and bifurcations, on average, lie between PR1 and PR2. Frequencies of normal modes of vibrations in PR3 are listed in Supporting Information in Table S1. The degenerate state energies of all 30 intermolecular modes, with harmonic frequencies up to 979.0 cm^–1^, lie below the rearrangement barriers.

**Figure 7:**
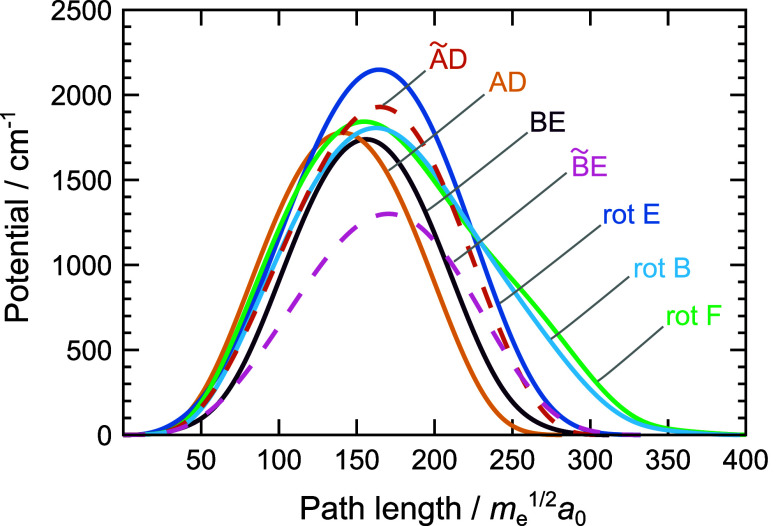
Potential energy curves along the minimum action paths for different rearrangement mechanisms in the water hexamer prism PR3, shown in Figure 6. See text for notation.

The TM elements in the GS and the low-lying vibrational states obtained using M-WKB, for each of the mechanisms discussed above, are listed in Table 2 and shown graphically in Figure 8. Convergence to better than 5% was achieved for the GS and the excited modes ν_1_–ν_5_, as in PR2. We, nevertheless, list the TM elements in modes ν_6_–ν_9_ as well, since, at least, a partial convergence was achieved for the dominant mechanisms. In Table 2, a heuristically defined uncertainty in the results is shown in the parentheses next to the TM element. It is the number of percentage points within which the TM elements lie as the number of discretization points is varied in the interval *N* = 300–1200 and the initial offset in the interval $\varepsilon =0.1m_{e}^{1/2}a_{0}-10m_{e}^{1/2}a_{0}$ . The numerical cause of the uncertainty is discussed above. Largest uncertainties are present in the TM elements for monomer rotations in the excited ν_6_–ν_9_. TM element for rot F in the excited mode ν_7_ did not converge, while those for the rot E and rot F in the excited mode ν_8_ are smaller than the value indicated in the table, with its sign uncertain. They are taken as zero in the calculation of TS patterns below.

**Table 2:** Tunneling Matrix Elements of the Water Hexamer Prism PR3 on MB-pol PES^24–26^ for Rearrangement Paths AD, ÃD, BE, B̃E, Rot B, Rot E and Rot F in the Vibrational Ground State and the 13 Lowest-Frequency Excited Vibrational Modes in cm^–1^

mode	*h*_AD_/10^–5^	$h_{ÃD}/10^{-7}$	*h*_BE_/10^–6^	$h_{\mathrm{B̃}E}/10^{-6}$
GS	–2.42	–8.81	–2.90	–5.30
1	–2.56	–9.41	–3.36	–7.68
2	–2.66	–12.7	–1.69	4.22
3	1.05	8.94	2.92	–5.68
4	–2.57	–7.89	–7.22	–5.61
5	2.33	7.43	–1.39	0.060
6	–15.7 (5)	–6.5 (5)	–11 (10)	–10 (40)
7	–2.70 (5)	–4.3 (5)	–7.1 (20)	–1.5 (60)
8	–1.20 (5)	–7.6 (10)	–1.7 (25)	–15 (50)
9	–2.8 (10)	2.0 (100)	–0.40 (25)	10 (10)

mode	*h*_rot B_/10^–6^	*h*_rot E_/10^–7^	*h*_rot F_/10^–7^
GS	–1.01	–5.36	–2.51
1	–2.27	–8.39	–3.65
2	1.51	–5.99	–5.42
3	1.51	–6.13	–4.74
4	–6.60	–9.31	–28.0
5	–14.0	–7.66	–27.3
6	–0.8 (50)	–9.0 (10)	–60 (50)
7	–5.6 (50)	–6.0 (10)	–
8	–0.8 (10)	<6	<2
9	–1.5 (100)	–1 (100)	–2 (100)

**Figure 8:**
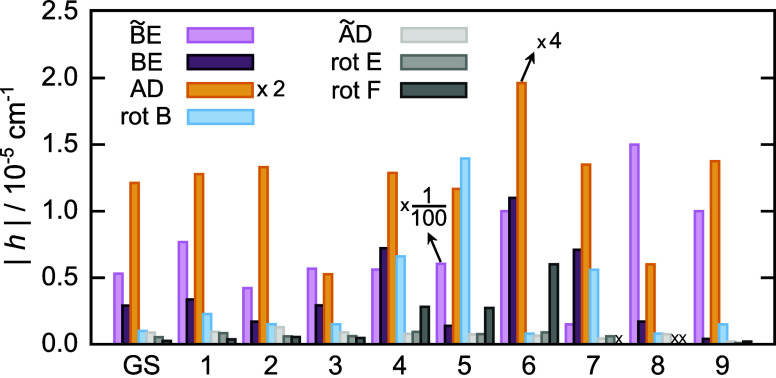
Tunneling matrix elements *h* of the water hexamer prism PR3 obtained using M-WKB on the MB-pol PES fort six rearrangement paths, AD, BE, ÃD, B̃E, rot B, rot E and rot F, shown in Figure 6 in the vibrational GS and the nine lowest-frequency excited modes, ν_1_–ν_9_, in cm^–1^.

The GS TS elements for flips and bifurcations all lie within 14% of those obtained in ref 45 using ring-polymer instanton theory. Discrepancy is somewhat larger for rot B (34%) and rot E (22%), which was converged to one significant digit in ref 45, and rot F, which is indeed smaller in our M-WKB calculations, was neglected. TM elements for monomer rotations were multiplied by two in Table 2, as above.

TM elements for flips and bifurcations in the excited states ν_1_–ν_5_ are similar in size to the corresponding GS TM elements. They are either enhanced or reduced by a factor that exceeds 2 only for AD in the excited ν_3_ (2.3× smaller), BE in ν_4_ (2.5× larger) and B̃E in ν_5_ (90× smaller, due to the cancellation of contributions of *F*- and *U*-terms in eq 5). There is no clear longitudinal mode for any of the mechanisms, as in PR2. In the excited modes ν_6_–ν_9_, variations are slightly greater and the factors range from the reduced by 6.5× for AD in ν_6_ to the enlarged by 7× for BE in ν_9_. TM elements for monomer rotations in the excited modes ν_1_–ν_5_ are all of larger size relative to the GS, as in PR2. The largest increases are for rot B in the excited ν_5_ (14×) and for rot F in the excited ν_4_ and ν_5_ (by ≈11×). In the excited modes ν_6_–ν_9_, we observe reductions as well as enhancements of TSs. The largest enhancement is for rot F in the excited mode ν_6_, by ≈20×. In general, the largest TM element sizes are, on average, for the mechanism AD, followed by B̃E. Looking at the potential profiles along their MAPs in Figure 7, the mechanism AD has the smallest tunneling path length, while B̃E has the smallest barrier. It is remarkable that the mechanism B̃E, although it involves a bifurcation which breaks two hydrogen bonds, has a smaller barrier than the double-flip mechanisms. The fact that the B̃E contribution does not dominate the TS pattern shows that the vibrational modes orthogonal to the MAP significantly affect the tunneling dynamics.

The neglected mechanisms in this study are ÃD̃, B̃Ẽ, rot A, rot C and rot D. All have MAP barrier heights above 3100 cm^–1^ and the GS TM elements are −4.9 × 10^–12^ cm^–1^, −6.4 × 10^–10^ cm^–1^, −7.2 × 10^–10^ cm^–1^, −7.2 × 10^–9^ cm^–1^ and −1.2 × 10^–10^ cm^–1^, respectively. The mechanism of quadruple flip BCEF decomposes into a two-step process of BE and AD, which is already included in the TM model.

Inclusion of any three mechanisms that involve flips or bifurcations of monomers A, D, B and E generates a group of 384 symmetry elements that we denote *G*_384_^′^ to distinguish from the group *G*_384_ of PR2. If all 7 mechanisms are included, any of the 384 permutational isomers can be reached in a maximum of 6 steps. The group again divides into 28 classes, which we define in Table S10 in Supporting Information. We constructed the character table of the group in Table S11 in Supporting Information and thereby defined the irrep names of the group. Symmetries of tunneling states, Γ_tun_, are obtained as Γ_tun_ = *A*_1_ ⊕ *A*_2_ ⊕ *A*_3_ ⊕ *A*_4_ ⊕ 2*E*_1_ ⊕ 2*E*_2_ ⊕ 2*E*_3_ ⊕ 3*T*_1_ ⊕ 3*T*_2_ ⊕ 3*T*_3_ ⊕ 3*T*_4_ ⊕ 3*T*_5_ ⊕ 3*T*_6_ ⊕ 3*T*_7_ ⊕ 3*T*_8_ ⊕ 3*T*_9_ ⊕ 3*T*_10_ ⊕ 3*T*_11_ ⊕ 3*T*_12_ ⊕ 2*G*_1_ ⊕ 6*I*_1_ ⊕ 6*I*_2_ ⊕ 6*I*_3_ ⊕ 6*I*_4_ ⊕ 6*I*_5_ ⊕ 6*I*_6_ ⊕ 6*I*_7_. Each degenerate vibrational level will therefore split into 90 delocalized states. The irrep names indicate state degeneracies in the same way as in PR2. Total wave function must be of Γ = *A*_3_ symmetry. Nuclear spin states span Γ_nuc_ = 138*A*_1_ ⊕ 111*A*_2_ ⊕ 1*A*_3_ ⊕ 240*E*_1_ ⊕ 11*E*_2_ ⊕ 45*T*_1_ ⊕ 36*T*_2_ ⊕ 45*T*_3_ ⊕ 36*T*_4_ ⊕ 45*T*_5_ ⊕ 36*T*_6_ ⊕ 6*T*_7_ ⊕ 3*T*_8_ ⊕ 6*T*_9_ ⊕ 3*T*_10_ ⊕ 6*T*_11_ ⊕ 3*T*_12_ ⊕ 16*G*_1_ ⊕ 243*I*_1_ ⊕ 81*I*_2_ ⊕ 27*I*_3_ ⊕ 27*I*_4_ ⊕ 27*I*_5_ ⊕ 9*I*_6_ ⊕ 3*I*_7_. There are no nuclear spin states of *A*_4_ and *E*_3_ symmetries. In the ground rotational state, vibrational states have the following statistical weights, Γ_vib_ = 1*A*_1_ ⊕ 138*A*_3_ ⊕ 111*A*_4_ ⊕ 11*E*_2_ ⊕ 240*E*_3_ ⊕ 6*T*_1_ ⊕ 3*T*_2_ ⊕ 6*T*_3_ ⊕ 3*T*_4_ ⊕ 6*T*_5_ ⊕ 3*T*_6_ ⊕ 45*T*_7_ ⊕ 36*T*_8_ ⊕ 45*T*_9_ ⊕ 36*T*_10_ ⊕ 45*T*_11_ ⊕ 36*T*_12_ ⊕ 8*G*_1_ ⊕ 3*I*_1_ ⊕ 9*I*_2_ ⊕ 27*I*_3_ ⊕ 27*I*_4_ ⊕ 26*I*_5_ ⊕ 81*I*_6_ ⊕ 243*I*_7_. Levels *A*_2_ and *E*_1_ are not populated. Again, assuming the dipole moment lies predominantly along the *C*_3_ axis of the prism, the symmetry operations in classes 21–28, see Table S10 in Supporting Information, change the direction of the dipole moment. The dipole moment is then of Γ_dip_ = *A*_2_ symmetry.

In the fully deuterated d12-prism PR3, the total wave function is of Γ = *A*_1_ symmetry. In the ground rotational state, Γ_vib_ = 7896*A*_1_ ⊕ 7680*A*_2_ ⊕ 138*A*_3_ ⊕ 111*A*_4_ ⊕ 15540*E*_1_ ⊕ 1956*E*_2_ ⊕ 240*E*_3_ ⊕ 5886*T*_1_ ⊕ 5778*T*_2_ ⊕ 5886*T*_3_ ⊕ 5778*T*_4_ ⊕ 5886*T*_5_ ⊕ 5778*T*_6_ ⊕ 1485*T*_7_ ⊕ 1431*T*_8_ ⊕ 1485*T*_9_ ⊕ 1431*T*_10_ ⊕ 1485*T*_11_ ⊕ 1431*T*_12_ ⊕ 3876*G*_1_ ⊕ 23328*I*_1_ ⊕ 11664*I*_2_ ⊕ 5832*I*_3_ ⊕ 5832*I*_4_ ⊕ 5832*I*_5_ ⊕ 2916*I*_6_ ⊕ 1458*I*_7_. All levels are populated. The d12-prism PR3 levels have not been computed here.

TM matrix is of dimension 384. Each row/column has 9 nonzero TM elements with 7 of them distinct, corresponding to mechanisms AD, BE, ÃD/AD̃, B̃E/BẼ, rot B, rot E and rot F. TM eigenvalues give tunneling state energies relative to the degenerate single-well state and eigenvectors give the state symmetries. Using TM elements in Table 2, we obtain 90 nondegenerate states per vibrational manifold and list them in Tables S12–S21 in Supporting Information. TS patterns for the GS and the excited vibrational modes ν_1_–ν_9_ are depicted in Figure 9 on the right-most panel (V).

**Figure 9:**
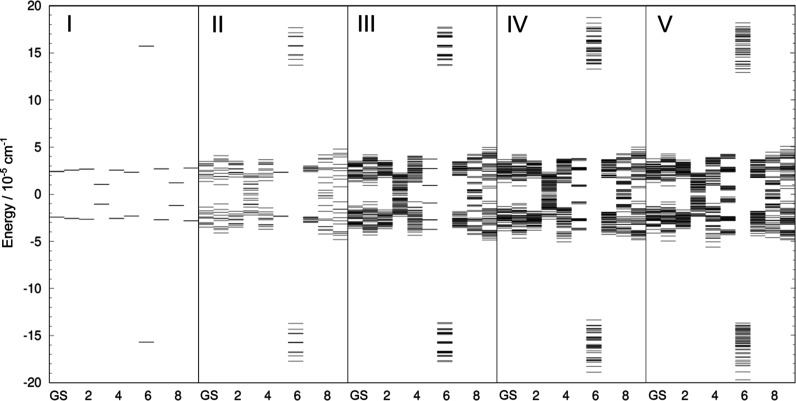
Tunneling splitting (TS) patterns of the water hexamer prism PR3 in the vibrational GS and the excited vibrational modes ν_1_–ν_9_. The rearrangement mechanisms included in the TS pattern estimates are the following: Panel I: AD, Panel II: AD and B̃E, Panel III: AD, B̃E and rot B, Panel IV: AD, B̃E, rot B and BE, Panel V: all 7 rearrangement mechanisms considered in the text and shown in Figure 6. See text for notation.

The TS pattern in Figure 9 reveals a doublet in the GS and the excited modes ν_1_–ν_4_, ν_6_ and ν_7_, with a spread of states in both branches. The doublet splitting is caused by the AD flip, as in PR1 and unlike PR2. In the GS, the AD flip alone produces a doublet that is 2|*h*_AD_| = 4.8 × 10^–5^ cm^–1^ wide. The overall GS TS width is 7.9 × 10^–5^ cm^–1^. The lower branch is spread over the width of 2.8 × 10^–5^ cm^–1^, while the upper branch is ≈10% narrower. Overall TS pattern widths in the excited states are similar to the GS TS width apart from the following. In the excited mode ν_6_, the overall width is 3.8 × 10^–4^ cm^–1^, an increase of 4.8× compared to the GS TS, with the two doublet branches having width 6.0 × 10^–5^ cm^–1^ (lower) and 5.2 × 10^–5^ cm^–1^ (upper). In the excited mode ν_3_, the TS width is reduced by a factor of 1.7 relative to the GS.

As stated above the doublet splittings in all states are caused by the mechanism AD. The mechanism AD alone produces the TS pattern shown in Figure 9, panel I. The overall appearance of the pattern is reproduced for most states. The next leading mechanism is B̃E/BẼ. Panel II in Figure 9 shows the TS pattern when mechanisms AD + B̃E/BẼ are included (and other TM elements set to zero). The appearance of the full TS pattern in panel V is now almost entirely reproduced, with the added doublet widths that the inclusion of B̃E/BẼ brings. The mechanism B̃E/BẼ has only a minor impact of the excited ν_5_ and ν_7_ patterns. A mismatch is seen for the excited mode ν_5_, which appears as a doublet, while panel V shows it should be a sextet. Incorporating rot B resolves the mismatch, as shown in panel III in Figure 9. Operations AD + rot B link 8 permutational isomers and form a sextet splitting having energies, $h_{AD}+h_{rot\;B}:-\sqrt{h_{AD}^{2}+h_{rot\;B}^{2}}:-\left|h_{AD}-h_{rot\;B}\right|:\left|h_{AD}-h_{rot\;B}\right|:$ $\sqrt{h_{AD}^{2}+h_{rot\;B}^{2}}:-\left(h_{AD}+h_{rot\;B}\right)$ , with the respective degeneracies 1:2:1:1:2:1. The addition of rot B significantly impacts the widths of doublet branches in ν_4_ and ν_7_. Panel IV shows the effect of adding BE. Its effect is mainly in adding widths in the excited modes ν_4_, ν_6_ and ν_7_.

Figure 10 compares the GS TS patterns in the hexamer prisms PR1, PR2 and PR3. The width of the GS in PR1 and PR3 are comparable in size. The largest splitting in PR1 and PR3 comes from a double flip (AD in both). The second leading contribution comes from a simultaneous bifurcation and a flip; on the same site in PR1 (ÃD) and on different sites in PR3 (B̃E), resulting in more nondegenerate states in the spectrum. Levels of PR1 that are shown in red in Figure 10 were obtained using instanton theory in ref 4 and used to interpret experiment. Third leading contribution in PR3 comes from another double flip mechanism, which is not present in PR1. The subsequent contributions in PR1 and PR3 come from monomer rotations. In PR1, rotations do not change the energies of the allowed dipole transitions.^48^ In PR2, flips/bifurcations are suppressed, and the largest contribution comes from monomer rotations, which have larger TM elements than in PR1 and PR3. The GS TS width of PR2 is significantly smaller and comparable to the doublet branch widths in PR1 and PR3. In the water decamer prism in both its forms, CWCW and CWCCW, double flips dominate the TS pattern, bifurcations have a smaller effect, while monomer rotations are negligible. The width of the CWCCW pattern is ≈2× wider than the CWCW. In the water hexamer prism, this factor is larger, 3.7, and compares two different mechanisms (double flip in CWCCW and rotation in CWCW). The ratio of TM elements for double flips in PR3 and PR2 is 27.

**Figure 10:**
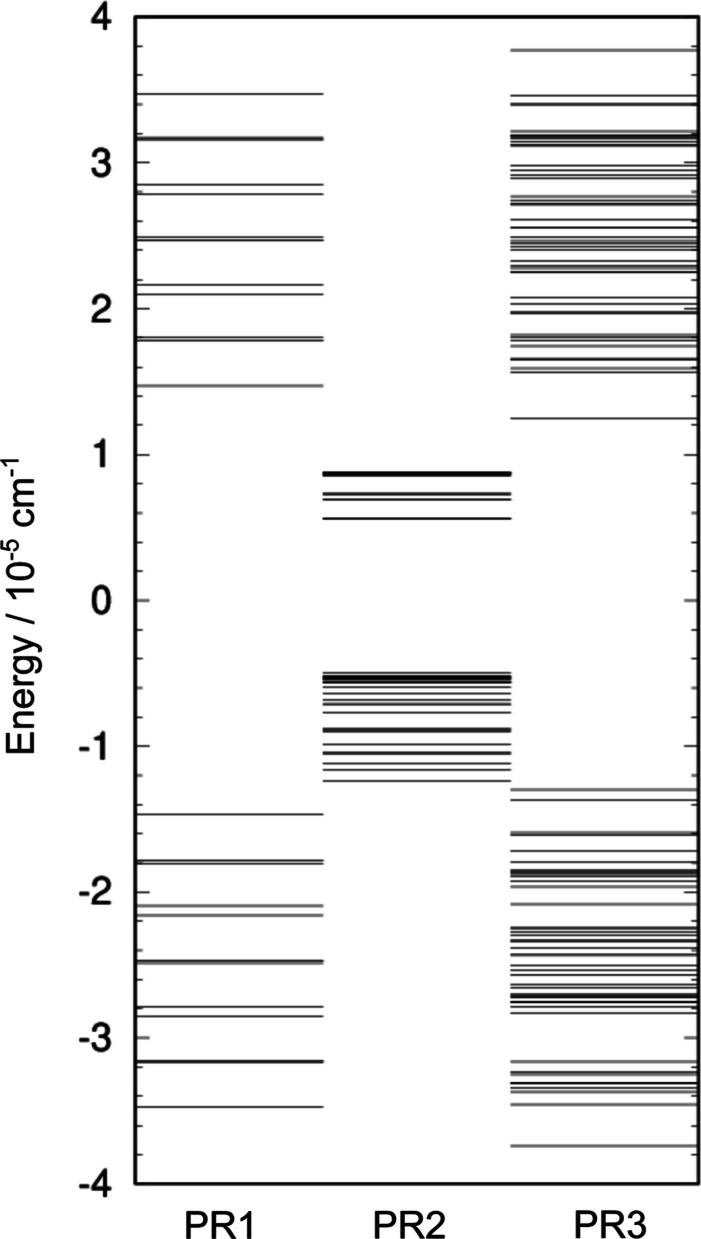
Ground-state tunneling splitting patterns of the water hexamer prisms PR1 (left), PR2 (mid) and PR3 (right). Energy levels in PR1 shown in red are obtained by omitting contributions from monomer rotations. These were used to interpret the experiment in ref 4.

## 4 Conclusions

We calculated the TS patterns in the GS and the low-lying excited vibrational states in the water hexamer prisms composed of the stacked water trimers in the CWCW, or PR2, and CWCCW, or PR3, forms, shown in Figure 1. For this purpose, we have employed the TM model in which we calculate the nearest-neighbor tunneling interactions using the recently developed semiclassical M-WKB method.^40,41^ The mechanisms responsible for the TS pattern formation were identified in ref 45 for the GS and we find that the same mechanisms, with varying strengths, participate in the formation of the TS patterns in the excited states. Relative state energies are obtained and, using group theory, their symmetries.

In summary, the calculated overall width of the TS pattern in PR3 is similar to that in PR1 ^48^ and a similar double-flip AD motion, shown in Figure 6a, is responsible. Other mechanisms that are relevant in the GS and the excited states are bifurcation B̃E, monomer rotation rot B, and the double-flip BE, shown in Figure 6d,e,b, respectively. The TS patterns of PR3 in the excited states ν_1_–ν_9_ are similar in all modes apart from the following. A qualitative change in the TS pattern is observed in the excited mode ν_5_, where AD and rot B mechanisms form a sextet of states. Moreover, in the excited mode ν_6_, we observe the largest increase of width, by 4.8×, relative to the GS. In PR2, double flips and bifurcations are suppressed relative to PR3 and to monomer rotations. In the GS and the excited modes ν_1_–ν_5_, the dominant rearrangement mechanism is monomer rotation rot B, shown in Figure 2d. Additional mechanism, rot F in Figure 2f, reveals itself in the spectrum in the excited modes ν_4_ and ν_5_, in which it forms a doublet of doublets. These states also exhibit the largest increases in the TS widths, by a factor of 3 and 9, respectively.

The M-WKB estimates are expected to give the right order of magnitude of the splittings and to identify the correct mechanisms responsible for the formation of the TS pattern. The theory cannot reliably predict the state energies or the order of states, as small changes in the sizes of TM elements would affect it. In the lowest-energy hexamer isomer, the prism PR1 in Figure 1, instanton theory has successfully explained the observed GS TS pattern in terms of the underlying rearrangement mechanisms.^4^ The splitting magnitudes were within a factor of 2 of both experiment and later numerically exact PIMD results.^10^ The M-WKB method uses the same approximations in a similar system and on the same PES, albeit in the vibrationally excited states, that cannot be treated using instanton theory or PIMD. Both M-WKB and instanton theory neglect the anharmonicity of the PES in directions perpendicular to the ‘optimal’ tunneling path (MAP). The M-WKB wave function is constructed using a harmonic oscillator wave function as the initial condition in eq 7 and is using its normalization factor, which introduces another source of error. Other approximations include the use of TM model, Herring formula and the neglect of overall rotations. For this system, in the deep tunneling regime, these approximations are probably not severe. The error is expected to be somewhat larger in the vibrationally excited states since the wave function spreads over larger configurational space in which the anharmonicity is neglected. To estimate the error arising from the anharmonicity, the theory would need to be extended to higher-order WKB terms.^61,62^ However, this would entail a substantial computational cost, as it requires evaluating higher-order expansion of the potential.

## Supplementary Material



## Notes

Frequencies of normal modes of vibration in the hexamer prisms PR2 and PR3; classes and character tables of the molecular symmetry groups of the hexamer prisms PR2 and PR3; tunneling splitting patterns with symmetries of vibrational states for the ground state and several excited states of the hexamer prisms PR2 and PR3; comparison of the ground-state tunneling matrix elements using MB-pol and using WHBB potential energy surfaces (PDF)
