# Supplementary material for: Tunneling Splittings in the Water Hexamer Prisms Composed of Stacked Water Trimers
Source: J Phys Chem A. 2025 Dec 11;129(51):11834–47. doi: 10.1021/acs.jpca.5c06786 (PMC12746455; doi:10.1021/acs.jpca.5c06786)
Supplement: Supplementary file 1 [file jp5c06786_si_001.pdf]

Supporting Information for publication "Tunneling Splittings in the Water Hexamer Prisms Composed of Stacked Water Trimers"

Nina Tokić[a] and Marko T. Cvitaš[a*]

[a] University of Zagreb Faculty of Science, Zagreb, Croatia;
E-mail: mcvitas.phy@pmf.hr

Table S1 Frequencies of normal modes of vibration in the hexamer prisms PR2 and PR3 in cm^{-1}.

Mode	PR2	PR3
1	61.8	63.1
2	67.6	66.6
3	69.3	78.1
4	86.5	87.1
5	92.1	103
6	161	157
7	174	180
8	190	192
9	199	197
10	202	203
11	222	211
12	243	240
13	255	252
14	266	264
15	285	283
16	367	372
17	383	400
18	406	413
19	424	429
20	461	468
21	465	473
22	486	475
23	540	520
24	588	550
25	598	608
26	649	679
27	692	701
28	784	785
29	919	902
30	943	979
31	1659	1658
32	1666	1668
33	1678	1676
34	1685	1679
35	1700	1699
36	1713	1710
37	3407	3396
38	3430	3421
39	3637	3631
40	3662	3650
41	3666	3662
42	3738	3723
43	3763	3774
44	3841	3834
45	3847	3845
46	3900	3901
47	3904	3902
48	3910	3908

Table S2 Definitions of classes in the G_{384} group of the hexamer prism PR2. Class name, number of elements in the class and a representative symmetry element of the class are in columns 1−3, respectively.

Class	size	element
1	1	E
2	6	(1 2)
3	6	(1 2)(3 4)
4	2	(1 2)(3 4)(5 6)
5	3	(1 2)(7 8)
6	6	(3 4)(7 8)
7	6	(1 2)(5 6)(7 8)
8	6	(1 2)(3 4)(7 8)
9	6	(3 4)(5 6)(7 8)
10	6	(1 2)(3 4)(5 6)(7 8)
11	3	(1 2)(3 4)(7 8)(9 10)
12	6	(1 2)(3 4)(7 8)(11 12)
13	6	(1 2)(3 4)(5 6)(7 8)(9 10)
14	1	(1 2)(3 4)(5 6)(7 8)(9 10)(11 12)
15	16	(ABC)(DEF)(1 3 5)(2 4 6)(7 9 11)(8 10 12)
16	32	(ABC)(DEF)(1 3 5)(2 4 6)(7 9 12 8 10 11)
17	16	(ABC)(DEF)(1 3 6 2 4 5)(7 9 12 8 10 11)
18	16	(ACB)(DFE)(1 5 3)(2 6 4)(7 11 9)(8 12 10)
19	32	(ACB)(DFE)(1 5 3)(2 6 4)(7 12 10 8 11 9)
20	16	(ACB)(DFE)(1 6 4 2 5 3)(7 12 10 8 11 9)
21	8	(AD)(BE)(CF)(1 7)(2 8)(3 9)(4 10)(5 11)(6 12)*
22	24	(AD)(BE)(CF)(1 7)(2 8)(3 9)(4 10)(5 12 6 11)*
23	24	(AD)(BE)(CF)(1 7)(2 8)(3 10 4 9)(5 12 6 11)*
24	8	(AD)(BE)(CF)(1 8 2 7)(3 10 4 9)(5 12 6 11)*
25	32	(AECDBF)(1 9 5 7 3 11)(2 10 6 8 4 12)*
26	32	(AECDBF)(1 9 5 7 3 12 2 10 6 8 4 11)*
27	32	(AFBDCE)(1 11 3 7 5 9)(2 12 4 8 6 10)*
28	32	(AFBDCE)(1 12 4 8 6 10 2 11 3 7 5 9)*

Table S3 Character table of the G_{384} group of the hexamer prism PR2. Class names are defined in Table S2.

	1	2	3	4	5	6	7	8	9	10	11	12	13	14	15	16	17	18	19	20	21	22	23	24	25	26	27	28
A_1^+	1	1	1	1	1	1	1	1	1	1	1	1	1	1	1	1	1	1	1	1	1	1	1	1	1	1	1	1
A_1^-	1	1	1	1	1	1	1	1	1	1	1	1	1	1	1	1	1	1	1	1	−1	−1	−1	−1	−1	−1	−1	−1
A_2^+	1	−1	1	−1	1	1	−1	−1	−1	1	1	1	−1	1	1	−1	1	1	−1	1	1	−1	1	−1	1	−1	1	−1
A_2^-	1	−1	1	−1	1	1	−1	−1	−1	1	1	1	−1	1	1	−1	1	1	−1	1	−1	1	−1	1	−1	1	−1	1
E_1^+	2	−2	2	−2	2	2	−2	−2	−2	2	2	2	−2	2	−1	1	−1	−1	1	−1	2	−2	2	−2	−1	1	−1	1
E_1^-	2	−2	2	−2	2	2	−2	−2	−2	2	2	2	−2	2	−1	1	−1	−1	1	−1	−2	2	−2	2	1	−1	1	−1
E_2^+	2	2	2	2	2	2	2	2	2	2	2	2	2	2	−1	−1	−1	−1	−1	−1	2	2	2	2	−1	−1	−1	−1
E_2^-	2	2	2	2	2	2	2	2	2	2	2	2	2	2	−1	−1	−1	−1	−1	−1	−2	−2	−2	−2	1	1	1	1
E_3	2	0	2	0	−2	−2	0	0	0	−2	2	2	0	−2	2	0	−2	2	0	−2	0	0	0	0	0	0	0	0
T_1^+	3	1	−1	−3	3	−1	1	1	−3	−1	3	−1	1	3	0	0	0	0	0	0	3	1	−1	−3	0	0	0	0
T_1^-	3	1	−1	−3	3	−1	1	1	−3	−1	3	−1	1	3	0	0	0	0	0	0	−3	−1	1	3	0	0	0	0
T_2^+	3	−1	−1	3	3	−1	−1	−1	3	−1	3	−1	−1	3	0	0	0	0	0	0	3	−1	−1	3	0	0	0	0
T_2^-	3	−1	−1	3	3	−1	−1	−1	3	−1	3	−1	−1	3	0	0	0	0	0	0	−3	1	1	−3	0	0	0	0
G_1	4	0	4	0	−4	−4	0	0	0	−4	4	4	0	−4	−2	0	2	−2	0	2	0	0	0	0	0	0	0	0
I_1	6	4	2	0	2	2	0	0	0	−2	−2	−2	−4	−6	0	0	0	0	0	0	0	0	0	0	0	0	0	0
I_2	6	2	2	6	−2	−2	−2	−2	−2	2	−2	−2	2	6	0	0	0	0	0	0	0	0	0	0	0	0	0	0
I_3	6	2	−2	−6	−2	2	−2	−2	2	−2	−2	2	2	6	0	0	0	0	0	0	0	0	0	0	0	0	0	0
I_4	6	0	−2	0	2	−2	4	−4	0	2	−2	2	0	−6	0	0	0	0	0	0	0	0	0	0	0	0	0	0
I_5	6	0	−2	0	2	−2	−4	4	0	2	−2	2	0	−6	0	0	0	0	0	0	0	0	0	0	0	0	0	0
I_6	6	0	−2	0	−6	2	0	0	0	2	6	−2	0	−6	0	0	0	0	0	0	0	0	0	0	0	0	0	0
I_7	6	−2	2	−6	−2	−2	2	2	2	2	−2	−2	−2	6	0	0	0	0	0	0	0	0	0	0	0	0	0	0
I_8	6	−2	−2	6	−2	2	2	2	−2	−2	−2	2	−2	6	0	0	0	0	0	0	0	0	0	0	0	0	0	0
I_9	6	−4	2	0	2	2	0	0	0	−2	−2	−2	4	−6	0	0	0	0	0	0	0	0	0	0	0	0	0	0

Table S4 Tunneling splitting pattern of the hexamer prism PR2 in its ground state.

State	Irrep	Energy level	State	Irrep	Energy level
39	A_1^-	-4.97680×10^{-6}	78	A_2^+	8.79720×10^{-6}
38	I_1	-5.14890×10^{-6}	77	I_9	8.75290×10^{-6}
37	I_3	-5.23681×10^{-6}	76	E_1^-	8.73310×10^{-6}
36	I_2	-5.26309×10^{-6}	75	I_8	8.72326×10^{-6}
35	I_5	-5.29238×10^{-6}	74	I_7	8.67331×10^{-6}
34	T_1^-	-5.29981×10^{-6}	73	I_6	8.67247×10^{-6}
33	T_1^+	-5.32170×10^{-6}	72	I_9	8.67159×10^{-6}
32	I_6	-5.32708×10^{-6}	71	I_8	8.61920×10^{-6}
31	I_8	-5.38904×10^{-6}	70	E_1^+	8.60490×10^{-6}
30	I_4	-5.40457×10^{-6}	69	I_9	8.58782×10^{-6}
29	I_7	-5.56604×10^{-6}	68	A_2^-	8.54080×10^{-6}
28	I_9	-5.56855×10^{-6}	67	T_2^-	7.33047×10^{-6}
27	G_1	-5.59685×10^{-6}	66	T_2^+	7.32438×10^{-6}
26	T_2^-	-5.60070×10^{-6}	65	I_5	7.32369×10^{-6}
25	T_2^+	-5.60089×10^{-6}	64	I_4	7.30144×10^{-6}
24	I_5	-5.60098×10^{-6}	63	I_3	7.29728×10^{-6}
23	I_7	-5.60740×10^{-6}	62	I_7	7.28236×10^{-6}
22	E_3	-5.62095×10^{-6}	61	I_9	7.28153×10^{-6}
21	I_4	-5.64204×10^{-6}	60	I_6	7.25852×10^{-6}
20	I_2	-5.64207×10^{-6}	59	I_8	7.25782×10^{-6}
19	I_1	-5.91434×10^{-6}	58	I_8	6.95171×10^{-6}
18	I_3	-6.34928×10^{-6}	57	I_6	6.95161×10^{-6}
17	E_2^+	-6.82290×10^{-6}	56	I_7	6.95086×10^{-6}
16	I_6	-7.10671×10^{-6}	55	I_9	6.95070×10^{-6}
15	I_2	-7.11686×10^{-6}	54	I_5	6.94613×10^{-6}
14	I_1	-7.72127×10^{-6}	53	T_2^+	6.94551×10^{-6}
13	I_3	-8.81287×10^{-6}	52	I_3	6.94544×10^{-6}
12	I_8	-8.82495×10^{-6}	51	I_4	6.94167×10^{-6}
11	I_4	-8.83837×10^{-6}	50	T_2^-	6.93923×10^{-6}
10	I_5	-8.95190×10^{-6}	49	I_2	5.64292×10^{-6}
9	T_1^+	-8.95863×10^{-6}	48	I_4	5.64186×10^{-6}
8	T_1^-	-8.97503×10^{-6}	47	E_3	5.62095×10^{-6}
7	I_1	-9.89375×10^{-6}	46	T_1^+	5.61133×10^{-6}
6	I_6	-1.04488×10^{-5}	45	I_1	5.60789×10^{-6}
5	E_2^-	-1.05151×10^{-5}	44	T_1^-	5.60584×10^{-6}
4	I_2	-1.05326×10^{-5}	43	I_7	5.60491×10^{-6}
3	I_3	-1.11818×10^{-5}	42	G_1	5.59685×10^{-6}
2	I_1	-1.16056×10^{-5}	41	I_5	5.57543×10^{-6}
1	A_1^+	-1.23612×10^{-5}	40	I_2	5.57373×10^{-6}

Table S5 Tunneling splitting pattern of the hexamer prism PR2 in the excited mode v_1.

State	Irrep	Energy level	State	Irrep	Energy level
39	E_3	-1.71155×10^{-5}	78	A_2^-	2.25858×10^{-5}
38	I_7	-1.71176×10^{-5}	77	I_9	2.24143×10^{-5}
37	I_9	-1.71249×10^{-5}	76	E_1^+	2.23529×10^{-5}
36	T_2^+	-1.71298×10^{-5}	75	I_8	2.22986×10^{-5}
35	I_5	-1.71299×10^{-5}	74	I_7	2.21515×10^{-5}
34	T_2^-	-1.71300×10^{-5}	73	I_9	2.21413×10^{-5}
33	I_4	-1.71328×10^{-5}	72	I_6	2.21411×10^{-5}
32	I_2	-1.71328×10^{-5}	71	I_8	2.19743×10^{-5}
31	G_1	-1.71336×10^{-5}	70	E_1^-	2.18871×10^{-5}
30	I_7	-1.71350×10^{-5}	69	I_9	2.18478×10^{-5}
29	T_1^+	-1.82801×10^{-5}	68	A_2^+	2.16542×10^{-5}
28	I_5	-1.82862×10^{-5}	67	T_2^+	1.98958×10^{-5}
27	T_1^-	-1.82977×10^{-5}	66	I_4	1.98861×10^{-5}
26	I_8	-1.83541×10^{-5}	65	T_2^-	1.98810×10^{-5}
25	I_4	-1.83567×10^{-5}	64	I_5	1.98294×10^{-5}
24	I_2	-1.86404×10^{-5}	63	I_3	1.98269×10^{-5}
23	I_6	-1.86831×10^{-5}	62	I_7	1.97333×10^{-5}
22	I_3	-1.87974×10^{-5}	61	I_6	1.97302×10^{-5}
21	I_1	-1.88190×10^{-5}	60	I_9	1.97209×10^{-5}
20	I_1	-1.90609×10^{-5}	59	I_8	1.97182×10^{-5}
19	I_3	-1.92192×10^{-5}	58	I_8	1.94912×10^{-5}
18	A_1^+	-1.94502×10^{-5}	57	I_6	1.94901×10^{-5}
17	I_6	-1.96257×10^{-5}	56	I_9	1.94806×10^{-5}
16	I_2	-1.96309×10^{-5}	55	I_7	1.94794×10^{-5}
15	E_2^-	-2.07851×10^{-5}	54	I_5	1.94204×10^{-5}
14	I_1	-2.08641×10^{-5}	53	I_3	1.94192×10^{-5}
13	I_8	-2.08881×10^{-5}	52	T_2^-	1.93690×10^{-5}
12	I_4	-2.08902×10^{-5}	51	I_4	1.93601×10^{-5}
11	T_1^-	-2.09531×10^{-5}	50	T_2^+	1.93539×10^{-5}
10	I_5	-2.09590×10^{-5}	49	I_2	1.71363×10^{-5}
9	T_1^+	-2.09694×10^{-5}	48	G_1	1.71336×10^{-5}
8	I_3	-2.16780×10^{-5}	47	I_4	1.71335×10^{-5}
7	I_1	-2.26997×10^{-5}	46	T_1^-	1.71308×10^{-5}
6	I_6	-2.30527×10^{-5}	45	T_1^+	1.71296×10^{-5}
5	I_2	-2.30932×10^{-5}	44	I_1	1.71294×10^{-5}
4	E_2^+	-2.34549×10^{-5}	43	I_7	1.71283×10^{-5}
3	I_3	-2.37915×10^{-5}	42	I_5	1.71253×10^{-5}
2	I_1	-2.41657×10^{-5}	41	I_2	1.71211×10^{-5}
1	A_1^-	-2.47898×10^{-5}	40	E_3	1.71155×10^{-5}

Table S6 Tunneling splitting pattern of the hexamer prism PR2 in the excited mode v_2.

State	Irrep	Energy level	State	Irrep	Energy level
39	E_3	-6.75299×10^{-6}	78	A_2^+	1.30088×10^{-5}
38	I_7	-6.75777×10^{-6}	77	I_9	1.29033×10^{-5}
37	T_2^-	-6.75915×10^{-6}	76	I_8	1.28214×10^{-5}
36	T_2^+	-6.75937×10^{-6}	75	E_1^-	1.27184×10^{-5}
35	I_5	-6.75999×10^{-6}	74	I_6	1.25276×10^{-5}
34	I_9	-6.76092×10^{-6}	73	I_7	1.25166×10^{-5}
33	I_4	-6.76266×10^{-6}	72	I_9	1.24639×10^{-5}
32	I_2	-6.76378×10^{-6}	71	E_1^+	1.21376×10^{-5}
31	I_7	-6.76869×10^{-6}	70	I_8	1.21147×10^{-5}
30	G_1	-6.77240×10^{-6}	69	I_9	1.19849×10^{-5}
29	I_4	-8.56046×10^{-6}	68	A_2^-	1.18472×10^{-5}
28	I_8	-8.56194×10^{-6}	67	I_5	1.06563×10^{-5}
27	I_3	-8.57414×10^{-6}	66	I_3	1.06562×10^{-5}
26	T_1^-	-8.57431×10^{-6}	65	T_2^-	1.06359×10^{-5}
25	T_1^+	-8.57545×10^{-6}	64	I_4	1.06349×10^{-5}
24	I_6	-8.57558×10^{-6}	63	T_2^+	1.06342×10^{-5}
23	I_5	-8.57676×10^{-6}	62	I_9	1.05494×10^{-5}
22	I_1	-8.58201×10^{-6}	61	I_8	1.05486×10^{-5}
21	I_2	-8.58388×10^{-6}	60	I_6	1.05295×10^{-5}
20	I_6	-1.05674×10^{-5}	59	I_7	1.05290×10^{-5}
19	I_2	-1.05678×10^{-5}	58	I_7	8.57419×10^{-6}
18	I_3	-1.05819×10^{-5}	57	I_9	8.57143×10^{-6}
17	I_1	-1.05827×10^{-5}	56	I_6	8.56082×10^{-6}
16	T_1^+	-1.06115×10^{-5}	55	I_8	8.55876×10^{-6}
15	I_5	-1.06115×10^{-5}	54	I_4	8.55507×10^{-6}
14	T_1^-	-1.06125×10^{-5}	53	T_2^+	8.55313×10^{-6}
13	I_8	-1.06256×10^{-5}	52	T_2^-	8.55128×10^{-6}
12	I_4	-1.06260×10^{-5}	51	I_3	8.53216×10^{-6}
11	A_1^-	-1.20784×10^{-5}	50	I_5	8.52990×10^{-6}
10	I_1	-1.21343×10^{-5}	49	G_1	6.77240×10^{-6}
9	I_3	-1.22021×10^{-5}	48	I_2	6.76855×10^{-6}
8	E_2^+	-1.22532×10^{-5}	47	I_7	6.76260×10^{-6}
7	I_1	-1.24417×10^{-5}	46	I_5	6.76210×10^{-6}
6	I_2	-1.24676×10^{-5}	45	I_1	6.76143×10^{-6}
5	I_6	-1.24749×10^{-5}	44	I_4	6.75911×10^{-6}
4	E_2^-	-1.26028×10^{-5}	43	T_1^+	6.75892×10^{-6}
3	I_3	-1.26862×10^{-5}	42	T_1^-	6.75879×10^{-6}
2	I_1	-1.27327×10^{-5}	41	I_2	6.75855×10^{-6}
1	A_1^+	-1.27776×10^{-5}	40	E_3	6.75299×10^{-6}

Table S7 Tunneling splitting pattern of the hexamer prism PR2 in the excited mode v_3.

State	Irrep	Energy level	State	Irrep	Energy level
39	E_3	-1.71021×10^{-5}	78	A_2^+	2.34984×10^{-5}
38	I_7	-1.71197×10^{-5}	77	I_9	2.29526×10^{-5}
37	I_4	-1.71378×10^{-5}	76	I_8	2.26303×10^{-5}
36	I_9	-1.71395×10^{-5}	75	E_1^-	2.23032×10^{-5}
35	I_2	-1.71400×10^{-5}	74	I_7	2.20468×10^{-5}
34	T_2^-	-1.71401×10^{-5}	73	I_6	2.20065×10^{-5}
33	T_2^+	-1.71428×10^{-5}	72	I_9	2.16781×10^{-5}
32	I_5	-1.71438×10^{-5}	71	I_8	2.07941×10^{-5}
31	G_1	-1.71564×10^{-5}	70	T_2^-	2.03633×10^{-5}
30	I_7	-1.71584×10^{-5}	69	I_4	2.03419×10^{-5}
29	T_1^-	-1.84612×10^{-5}	68	T_2^+	2.03253×10^{-5}
28	I_5	-1.84783×10^{-5}	67	I_5	2.02842×10^{-5}
27	T_1^+	-1.84996×10^{-5}	66	I_3	2.02818×10^{-5}
26	I_8	-1.85345×10^{-5}	65	I_9	2.00551×10^{-5}
25	I_4	-1.85369×10^{-5}	64	E_1^+	1.99128×10^{-5}
24	I_2	-1.87349×10^{-5}	63	I_6	1.91647×10^{-5}
23	I_1	-1.87350×10^{-5}	62	I_7	1.91520×10^{-5}
22	I_3	-1.87352×10^{-5}	61	I_8	1.88204×10^{-5}
21	I_6	-1.87417×10^{-5}	60	A_2^-	1.87176×10^{-5}
20	I_1	-1.91406×10^{-5}	59	I_9	1.86648×10^{-5}
19	I_3	-1.91791×10^{-5}	58	I_9	1.82209×10^{-5}
18	I_2	-1.92117×10^{-5}	57	I_8	1.82164×10^{-5}
17	I_6	-1.92323×10^{-5}	56	I_6	1.81784×10^{-5}
16	I_8	-1.97107×10^{-5}	55	I_7	1.81534×10^{-5}
15	I_4	-1.97127×10^{-5}	54	I_5	1.79638×10^{-5}
14	T_1^+	-1.97509×10^{-5}	53	I_3	1.79616×10^{-5}
13	I_5	-1.97675×10^{-5}	52	T_2^+	1.79255×10^{-5}
12	T_1^-	-1.97879×10^{-5}	51	I_4	1.79034×10^{-5}
11	A_1^-	-1.98936×10^{-5}	50	T_2^-	1.78849×10^{-5}
10	I_1	-2.04194×10^{-5}	49	I_2	1.71583×10^{-5}
9	E_2^+	-2.05008×10^{-5}	48	G_1	1.71564×10^{-5}
8	I_3	-2.07650×10^{-5}	47	T_1^+	1.71425×10^{-5}
7	I_1	-2.12835×10^{-5}	46	I_4	1.71422×10^{-5}
6	I_6	-2.13756×10^{-5}	45	I_7	1.71419×10^{-5}
5	I_2	-2.14059×10^{-5}	44	I_5	1.71415×10^{-5}
4	E_2^-	-2.17152×10^{-5}	43	T_1^-	1.71411×10^{-5}
3	I_3	-2.17801×10^{-5}	42	I_1	1.71379×10^{-5}
2	I_1	-2.19914×10^{-5}	41	I_2	1.71181×10^{-5}
1	A_1^+	-2.23224×10^{-5}	40	E_3	1.71021×10^{-5}

Table S8 Tunneling splitting pattern of the hexamer prism PR2 in the excited mode v_4.

State	Irrep	Energy level	State	Irrep	Energy level
39	I_7	-1.76191×10^{-5}	78	A_2^-	6.20358×10^{-5}
38	I_9	-1.76246×10^{-5}	77	I_9	6.18449×10^{-5}
37	T_2^-	-1.76260×10^{-5}	76	I_8	6.17003×10^{-5}
36	G_1	-1.76260×10^{-5}	75	E_1^+	6.16129×10^{-5}
35	T_2^+	-1.76262×10^{-5}	74	I_6	6.12548×10^{-5}
34	I_5	-1.76266×10^{-5}	73	I_7	6.12533×10^{-5}
33	I_4	-1.76405×10^{-5}	72	I_9	6.12174×10^{-5}
32	I_2	-1.76412×10^{-5}	71	E_1^-	6.07671×10^{-5}
31	I_7	-1.76432×10^{-5}	70	I_8	6.07309×10^{-5}
30	E_3	-1.76618×10^{-5}	69	I_9	6.05572×10^{-5}
29	I_8	-2.15785×10^{-5}	68	A_2^+	6.03442×10^{-5}
28	I_4	-2.15800×10^{-5}	67	I_5	5.72282×10^{-5}
27	I_6	-2.15806×10^{-5}	66	I_3	5.72276×10^{-5}
26	I_3	-2.15821×10^{-5}	65	T_2^-	5.72267×10^{-5}
25	I_5	-2.15849×10^{-5}	64	T_2^+	5.72264×10^{-5}
24	T_1^+	-2.15873×10^{-5}	63	I_4	5.72260×10^{-5}
23	I_2	-2.15873×10^{-5}	62	I_9	5.71750×10^{-5}
22	T_1^-	-2.15883×10^{-5}	61	I_8	5.71743×10^{-5}
21	I_1	-2.15902×10^{-5}	60	I_7	5.71612×10^{-5}
20	I_1	-5.69962×10^{-5}	59	I_6	5.71607×10^{-5}
19	I_3	-5.69980×10^{-5}	58	I_7	2.15916×10^{-5}
18	I_6	-5.70339×10^{-5}	57	I_4	2.15911×10^{-5}
17	I_2	-5.70342×10^{-5}	56	I_9	2.15900×10^{-5}
16	I_8	-5.72333×10^{-5}	55	T_2^+	2.15898×10^{-5}
15	I_4	-5.72334×10^{-5}	54	T_2^-	2.15893×10^{-5}
14	T_1^-	-5.72363×10^{-5}	53	I_6	2.15870×10^{-5}
13	I_5	-5.72365×10^{-5}	52	I_3	2.15864×10^{-5}
12	T_1^+	-5.72369×10^{-5}	51	I_8	2.15863×10^{-5}
11	A_1^-	-5.93318×10^{-5}	50	I_5	2.15856×10^{-5}
10	I_1	-5.99987×10^{-5}	49	E_3	1.76618×10^{-5}
9	E_2^+	-6.02609×10^{-5}	48	I_2	1.76496×10^{-5}
8	I_3	-6.04589×10^{-5}	47	I_4	1.76368×10^{-5}
7	I_1	-6.12991×10^{-5}	46	I_7	1.76363×10^{-5}
6	I_6	-6.13880×10^{-5}	45	I_1	1.76352×10^{-5}
5	I_2	-6.13908×10^{-5}	44	T_1^-	1.76347×10^{-5}
4	E_2^-	-6.21191×10^{-5}	43	T_1^+	1.76342×10^{-5}
3	I_3	-6.21550×10^{-5}	42	I_5	1.76341×10^{-5}
2	I_1	-6.25110×10^{-5}	41	G_1	1.76260×10^{-5}
1	A_1^+	-6.30482×10^{-5}	40	I_2	1.76239×10^{-5}

Table S9 Tunneling splitting pattern of the hexamer prism PR2 in the excited mode v_5.

State	Irrep	Energy level	State	Irrep	Energy level
39	G_1	-1.80558×10^{-4}	78	A_2^+	1.96777×10^{-4}
38	I_7	-1.80558×10^{-4}	77	I_9	1.96485×10^{-4}
37	I_9	-1.80561×10^{-4}	76	I_8	1.96284×10^{-4}
36	T_2^+	-1.80562×10^{-4}	75	E_1^-	1.96113×10^{-4}
35	I_5	-1.80562×10^{-4}	74	I_6	1.95711×10^{-4}
34	T_2^-	-1.80562×10^{-4}	73	I_7	1.95703×10^{-4}
33	I_4	-1.80563×10^{-4}	72	I_9	1.95568×10^{-4}
32	I_2	-1.80563×10^{-4}	71	I_8	1.94857×10^{-4}
31	I_7	-1.80565×10^{-4}	70	E_1^+	1.94786×10^{-4}
30	E_3	-1.80570×10^{-4}	69	I_9	1.94528×10^{-4}
29	T_1^-	-1.82442×10^{-4}	68	A_2^-	1.94123×10^{-4}
28	I_5	-1.82442×10^{-4}	67	I_5	1.93253×10^{-4}
27	T_1^+	-1.82444×10^{-4}	66	I_3	1.93253×10^{-4}
26	I_8	-1.82469×10^{-4}	65	T_2^+	1.93244×10^{-4}
25	I_4	-1.82470×10^{-4}	64	I_4	1.93243×10^{-4}
24	I_2	-1.82498×10^{-4}	63	T_2^-	1.93243×10^{-4}
23	I_1	-1.82500×10^{-4}	62	I_9	1.93004×10^{-4}
22	I_6	-1.82521×10^{-4}	61	I_8	1.93002×10^{-4}
21	I_3	-1.82523×10^{-4}	60	I_6	1.92983×10^{-4}
20	A_1^+	-1.91417×10^{-4}	59	I_7	1.92982×10^{-4}
19	I_1	-1.91577×10^{-4}	58	I_7	1.82777×10^{-4}
18	I_3	-1.91739×10^{-4}	57	I_9	1.82776×10^{-4}
17	I_6	-1.92170×10^{-4}	56	T_2^-	1.82769×10^{-4}
16	I_2	-1.92177×10^{-4}	55	I_4	1.82769×10^{-4}
15	E_2^-	-1.93433×10^{-4}	54	T_2^+	1.82769×10^{-4}
14	I_1	-1.93527×10^{-4}	53	I_6	1.82768×10^{-4}
13	I_8	-1.93542×10^{-4}	52	I_8	1.82768×10^{-4}
12	I_4	-1.93542×10^{-4}	51	I_3	1.82759×10^{-4}
11	T_1^+	-1.93568×10^{-4}	50	I_5	1.82759×10^{-4}
10	I_5	-1.93569×10^{-4}	49	E_3	1.80570×10^{-4}
9	T_1^-	-1.93571×10^{-4}	48	I_2	1.80566×10^{-4}
8	I_3	-1.94687×10^{-4}	47	I_1	1.80563×10^{-4}
7	I_1	-1.96225×10^{-4}	46	I_4	1.80562×10^{-4}
6	I_6	-1.96771×10^{-4}	45	T_1^+	1.80562×10^{-4}
5	I_2	-1.96787×10^{-4}	44	T_1^-	1.80562×10^{-4}
4	E_2^+	-1.97467×10^{-4}	43	I_7	1.80562×10^{-4}
3	I_3	-1.97963×10^{-4}	42	I_5	1.80562×10^{-4}
2	I_1	-1.98533×10^{-4}	41	I_2	1.80558×10^{-4}
1	A_1^-	-1.99483×10^{-4}	40	G_1	1.80558×10^{-4}

Table S10 Definitions of classes in the G'_{384} group of the hexamer prism PR3. Class name, number of elements in the class and a representative symmetry element of the class are in columns 1−3, respectively.

Class	size	element
1	1	E
2	6	(1 2)
3	6	(1 2)(3 4)
4	2	(1 2)(3 4)(5 6)
5	3	(1 2)(7 8)
6	3	(5 6)(7 8)
7	6	(1 2)(5 6)(7 8)
8	3	(3 4)(7 8)
9	6	(1 2)(3 4)(7 8)
10	6	(3 4)(5 6)(7 8)
11	6	(1 2)(3 4)(5 6)(7 8)
12	3	(1 2)(5 6)(7 8)(9 10)
13	3	(1 2)(3 4)(7 8)(9 10)
14	3	(3 4)(5 6)(7 8)(9 10)
15	6	(1 2)(3 4)(5 6)(7 8)(9 10)
16	1	(1 2)(3 4)(5 6)(7 8)(9 10)(11 12)
17	32	(ABC)(DEF)(1 3 5)(2 4 6)(7 9 11)(8 10 12)
18	32	(ABC)(DEF)(1 3 5)(2 4 6)(7 9 12 8 10 11)
19	32	(ABC)(DEF)(1 3 6 2 4 5)(7 9 11)(8 10 12)
20	32	(ABC)(DEF)(1 3 6 2 4 5)(7 9 12 8 10 11)
21	24	(AE)(BD)(CF)(1 9)(2 10)(3 7)(4 8)(5 11)(6 12)
22	24	(AE)(BD)(CF)(1 9)(2 10)(3 7)(4 8)(5 12 6 11)
23	24	(AE)(BD)(CF)(1 9)(2 10)(3 8 4 7)(5 11)(6 12)
24	24	(AE)(BD)(CF)(1 9)(2 10)(3 8 4 7)(5 12 6 11)
25	24	(AE)(BD)(CF)(1 10 2 9)(3 7)(4 8)(5 11)(6 12)
26	24	(AE)(BD)(CF)(1 10 2 9)(3 7)(4 8)(5 12 6 11)
27	24	(AE)(BD)(CF)(1 10 2 9)(3 8 4 7)(5 11)(6 12)
28	24	(AE)(BD)(CF)(1 10 2 9)(3 8 4 7)(5 12 6 11)

Table S11 Character table of the G'_{384} group of the hexamer prism PR3. Class names are defined in Table S10.

	1	2	3	4	5	6	7	8	9	10	11	12	13	14	15	16	17	18	19	20	21	22	23	24	25	26	27	28
A_1	1	1	1	1	1	1	1	1	1	1	1	1	1	1	1	1	1	1	1	1	1	1	1	1	1	1	1	1
A_2	1	1	1	1	1	1	1	1	1	1	1	1	1	1	1	1	1	1	1	1	−1	−1	−1	−1	−1	−1	−1	−1
A_3	1	−1	1	−1	1	1	−1	1	−1	−1	1	1	1	1	−1	1	1	−1	−1	1	1	−1	−1	1	−1	1	1	−1
A_4	1	−1	1	−1	1	1	−1	1	−1	−1	1	1	1	1	−1	1	1	−1	−1	1	−1	1	1	−1	1	−1	−1	1
E_1	2	2	2	2	2	2	2	2	2	2	2	2	2	2	2	2	−1	−1	−1	−1	0	0	0	0	0	0	0	0
E_2	2	0	2	0	−2	−2	0	−2	0	0	−2	2	2	2	0	−2	2	0	0	−2	0	0	0	0	0	0	0	0
E_3	2	−2	2	−2	2	2	−2	2	−2	−2	2	2	2	2	−2	2	−1	1	1	−1	0	0	0	0	0	0	0	0
T_1	3	1	−1	−3	3	−1	1	−1	1	−3	−1	−1	3	−1	1	3	0	0	0	0	1	−1	1	−1	1	−1	1	−1
T_2	3	1	−1	−3	3	−1	1	−1	1	−3	−1	−1	3	−1	1	3	0	0	0	0	−1	1	−1	1	−1	1	−1	1
T_3	3	1	−1	−3	−1	3	1	−1	−3	1	−1	3	−1	−1	1	3	0	0	0	0	1	1	1	1	−1	−1	−1	−1
T_4	3	1	−1	−3	−1	3	1	−1	−3	1	−1	3	−1	−1	1	3	0	0	0	0	−1	−1	−1	−1	1	1	1	1
T_5	3	1	−1	−3	−1	−1	−3	3	1	1	−1	−1	−1	3	1	3	0	0	0	0	1	1	−1	−1	1	1	−1	−1
T_6	3	1	−1	−3	−1	−1	−3	3	1	1	−1	−1	−1	3	1	3	0	0	0	0	−1	−1	1	1	−1	−1	1	1
T_7	3	−1	−1	3	3	−1	−1	−1	−1	3	−1	−1	3	−1	−1	3	0	0	0	0	1	1	−1	−1	−1	−1	1	1
T_8	3	−1	−1	3	3	−1	−1	−1	−1	3	−1	−1	3	−1	−1	3	0	0	0	0	−1	−1	1	1	1	1	−1	−1
T_9	3	−1	−1	3	−1	3	−1	−1	3	−1	−1	3	−1	−1	−1	3	0	0	0	0	1	−1	−1	1	1	−1	−1	1
T_{10}	3	−1	−1	3	−1	3	−1	−1	3	−1	−1	3	−1	−1	−1	3	0	0	0	0	−1	1	1	−1	−1	1	1	−1
T_{11}	3	−1	−1	3	−1	−1	3	3	−1	−1	−1	−1	−1	3	−1	3	0	0	0	0	1	−1	1	−1	−1	1	−1	1
T_{12}	3	−1	−1	3	−1	−1	3	3	−1	−1	−1	−1	−1	3	−1	3	0	0	0	0	−1	1	−1	1	1	−1	1	−1
G_1	4	0	4	0	−4	−4	0	−4	0	0	−4	4	4	4	0	−4	−2	0	0	2	0	0	0	0	0	0	0	0
I_1	6	4	2	0	2	2	0	2	0	0	−2	−2	−2	−2	−4	−6	0	0	0	0	0	0	0	0	0	0	0	0
I_2	6	2	2	6	−2	−2	−2	−2	−2	−2	2	−2	−2	−2	2	6	0	0	0	0	0	0	0	0	0	0	0	0
I_3	6	0	−2	0	2	2	0	−6	0	0	2	−2	−2	6	0	−6	0	0	0	0	0	0	0	0	0	0	0	0
I_4	6	0	−2	0	2	−6	0	2	0	0	2	6	−2	−2	0	−6	0	0	0	0	0	0	0	0	0	0	0	0
I_5	6	0	−2	0	−6	2	0	2	0	0	2	−2	6	−2	0	−6	0	0	0	0	0	0	0	0	0	0	0	0
I_6	6	−2	2	−6	−2	−2	2	−2	2	2	2	−2	−2	−2	−2	6	0	0	0	0	0	0	0	0	0	0	0	0
I_7	6	−4	2	0	2	2	0	2	0	0	−2	−2	−2	−2	4	−6	0	0	0	0	0	0	0	0	0	0	0	0

Table S12 Tunneling splitting pattern of the hexamer prism PR3 in its ground state.

State	Irrep	Energy level	State	Irrep	Energy level
45	A_3	-1.29410×10^{-5}	90	A_2	3.76650×10^{-5}
44	T_1	-1.36987×10^{-5}	89	I_1	3.45700×10^{-5}
43	I_4	-1.58813×10^{-5}	88	T_8	3.39708×10^{-5}
42	I_3	-1.60856×10^{-5}	87	T_2	3.21051×10^{-5}
41	T_8	-1.71662×10^{-5}	86	I_3	3.18346×10^{-5}
40	I_7	-1.79411×10^{-5}	85	I_4	3.17967×10^{-5}
39	T_9	-1.84481×10^{-5}	84	T_1	3.16786×10^{-5}
38	I_6	-1.85847×10^{-5}	83	T_6	3.16508×10^{-5}
37	T_{11}	-1.86544×10^{-5}	82	I_2	3.14124×10^{-5}
36	T_6	-1.88351×10^{-5}	81	T_4	3.11919×10^{-5}
35	I_1	-1.88611×10^{-5}	80	I_7	3.11259×10^{-5}
34	I_2	-1.92143×10^{-5}	79	T_9	2.97706×10^{-5}
33	T_4	-1.96096×10^{-5}	78	I_6	2.94464×10^{-5}
32	I_7	-2.07858×10^{-5}	77	T_{11}	2.90884×10^{-5}
31	I_5	-2.24260×10^{-5}	76	E_3	2.89176×10^{-5}
30	T_7	-2.26786×10^{-5}	75	I_3	2.76423×10^{-5}
29	G_1	-2.28893×10^{-5}	74	I_4	2.73996×10^{-5}
28	I_5	-2.32705×10^{-5}	73	I_5	2.71811×10^{-5}
27	T_{11}	-2.33750×10^{-5}	72	E_2	2.71009×10^{-5}
26	I_6	-2.37678×10^{-5}	71	T_4	2.60828×10^{-5}
25	T_9	-2.42635×10^{-5}	70	I_1	2.55093×10^{-5}
24	E_1	-2.42867×10^{-5}	69	I_2	2.54854×10^{-5}
23	T_3	-2.50037×10^{-5}	68	T_6	2.48493×10^{-5}
22	E_3	-2.53236×10^{-5}	67	T_{12}	2.45857×10^{-5}
21	I_2	-2.56802×10^{-5}	66	T_8	2.44544×10^{-5}
20	T_5	-2.63390×10^{-5}	65	I_6	2.42195×10^{-5}
19	I_3	-2.65369×10^{-5}	64	T_{10}	2.39960×10^{-5}
18	I_4	-2.69439×10^{-5}	63	I_5	2.32705×10^{-5}
17	T_{10}	-2.70895×10^{-5}	62	G_1	2.28893×10^{-5}
16	E_2	-2.71009×10^{-5}	61	I_7	2.27386×10^{-5}
15	I_5	-2.71811×10^{-5}	60	I_5	2.24260×10^{-5}
14	I_6	-2.74554×10^{-5}	59	E_1	2.06927×10^{-5}
13	I_1	-2.75317×10^{-5}	58	I_7	2.03116×10^{-5}
12	T_{12}	-2.77828×10^{-5}	57	I_6	1.97361×10^{-5}
11	I_7	-2.82612×10^{-5}	56	T_{12}	1.97321×10^{-5}
10	T_2	-3.15699×10^{-5}	55	T_{10}	1.96285×10^{-5}
9	I_4	-3.22584×10^{-5}	54	T_5	1.81739×10^{-5}
8	I_3	-3.24532×10^{-5}	53	T_7	1.80353×10^{-5}
7	T_7	-3.30217×10^{-5}	52	I_2	1.77727×10^{-5}
6	T_5	-3.30939×10^{-5}	51	T_3	1.73893×10^{-5}
5	I_2	-3.33700×10^{-5}	50	A_4	1.65350×10^{-5}
4	T_3	-3.36445×10^{-5}	49	I_1	1.64675×10^{-5}
3	T_1	-3.45150×10^{-5}	48	I_4	1.58872×10^{-5}
2	I_1	-3.73420×10^{-5}	47	I_3	1.55988×10^{-5}
1	A_1	-4.12590×10^{-5}	46	T_2	1.24059×10^{-5}

Table S13 Tunneling splitting pattern of the hexamer prism PR3 in the excited mode v_1.

State	Irrep	Energy level	State	Irrep	Energy level
45	A_3	-8.24400×10^{-6}	90	A_2	4.27280×10^{-5}
44	T_1	-1.08382×10^{-5}	89	T_8	4.05773×10^{-5}
43	I_4	-1.42623×10^{-5}	88	I_1	3.89021×10^{-5}
42	I_3	-1.46700×10^{-5}	87	I_7	3.73404×10^{-5}
41	I_7	-1.60023×10^{-5}	86	I_3	3.72217×10^{-5}
40	T_9	-1.70332×10^{-5}	85	I_4	3.69972×10^{-5}
39	T_8	-1.72017×10^{-5}	84	T_1	3.68665×10^{-5}
38	I_6	-1.76781×10^{-5}	83	T_2	3.60671×10^{-5}
37	T_{11}	-1.80856×10^{-5}	82	T_6	3.56002×10^{-5}
36	T_6	-1.83069×10^{-5}	81	T_9	3.52019×10^{-5}
35	I_1	-1.88166×10^{-5}	80	I_2	3.50191×10^{-5}
34	I_2	-1.93357×10^{-5}	79	E_3	3.49567×10^{-5}
33	T_4	-2.04056×10^{-5}	78	I_6	3.45079×10^{-5}
32	I_7	-2.18329×10^{-5}	77	T_4	3.44697×10^{-5}
31	I_5	-2.28467×10^{-5}	76	T_{11}	3.37034×10^{-5}
30	T_7	-2.34483×10^{-5}	75	I_3	3.03258×10^{-5}
29	T_{11}	-2.38618×10^{-5}	74	I_4	2.99459×10^{-5}
28	G_1	-2.41199×10^{-5}	73	I_5	2.93737×10^{-5}
27	I_5	-2.49550×10^{-5}	72	E_2	2.89796×10^{-5}
26	I_6	-2.49750×10^{-5}	71	T_4	2.86639×10^{-5}
25	T_3	-2.56186×10^{-5}	70	I_1	2.73891×10^{-5}
24	T_9	-2.64127×10^{-5}	69	I_2	2.71249×10^{-5}
23	I_2	-2.73808×10^{-5}	68	T_{12}	2.66979×10^{-5}
22	E_1	-2.77893×10^{-5}	67	T_8	2.63004×10^{-5}
21	E_3	-2.80087×10^{-5}	66	I_7	2.57876×10^{-5}
20	I_3	-2.84198×10^{-5}	65	I_6	2.55480×10^{-5}
19	E_2	-2.89796×10^{-5}	64	T_6	2.54347×10^{-5}
18	T_5	-2.90762×10^{-5}	63	T_{10}	2.50042×10^{-5}
17	I_4	-2.93463×10^{-5}	62	I_5	2.49550×10^{-5}
16	I_5	-2.93737×10^{-5}	61	G_1	2.41199×10^{-5}
15	T_{10}	-2.95682×10^{-5}	60	I_5	2.28467×10^{-5}
14	I_6	-3.04415×10^{-5}	59	E_1	2.08413×10^{-5}
13	T_{12}	-3.11705×10^{-5}	58	I_7	2.02878×10^{-5}
12	I_1	-3.15396×10^{-5}	57	I_6	1.99868×10^{-5}
11	I_7	-3.16847×10^{-5}	56	T_{10}	1.97560×10^{-5}
10	T_2	-3.65668×10^{-5}	55	T_7	1.97032×10^{-5}
9	I_4	-3.80088×10^{-5}	54	T_{12}	1.96646×10^{-5}
8	I_3	-3.84619×10^{-5}	53	T_5	1.79378×10^{-5}
7	T_5	-3.85376×10^{-5}	52	I_2	1.68143×10^{-5}
6	T_7	-3.89829×10^{-5}	51	T_3	1.57488×10^{-5}
5	I_2	-3.91897×10^{-5}	50	A_4	1.51920×10^{-5}
4	T_3	-3.98062×10^{-5}	49	I_4	1.46743×10^{-5}
3	T_1	-4.12203×10^{-5}	48	I_1	1.46386×10^{-5}
2	I_1	-4.44696×10^{-5}	47	I_3	1.40042×10^{-5}
1	A_1	-4.96760×10^{-5}	46	T_2	8.74367×10^{-6}

Table S14 Tunneling splitting pattern of the hexamer prism PR3 in the excited mode ν_2.

State	Irrep	Energy level	State	Irrep	Energy level
45	T_7	-1.66950×10^{-5}	90	T_2	3.61901×10^{-5}
44	I_7	-1.92710×10^{-5}	89	I_4	3.39653×10^{-5}
43	I_3	-1.99833×10^{-5}	88	A_4	3.38210×10^{-5}
42	I_4	-2.04196×10^{-5}	87	I_1	3.38000×10^{-5}
41	T_{12}	-2.11425×10^{-5}	86	T_8	3.37924×10^{-5}
40	E_3	-2.12920×10^{-5}	85	E_1	3.34053×10^{-5}
39	A_1	-2.20210×10^{-5}	84	I_3	3.33200×10^{-5}
38	I_6	-2.20986×10^{-5}	83	I_7	3.30439×10^{-5}
37	T_3	-2.22163×10^{-5}	82	T_{12}	3.25604×10^{-5}
36	T_9	-2.24188×10^{-5}	81	I_6	3.20700×10^{-5}
35	T_1	-2.28048×10^{-5}	80	T_{10}	3.14873×10^{-5}
34	I_2	-2.30389×10^{-5}	79	T_3	3.11082×10^{-5}
33	T_{10}	-2.31347×10^{-5}	78	T_6	3.09107×10^{-5}
32	I_1	-2.33181×10^{-5}	77	I_2	3.06077×10^{-5}
31	T_5	-2.39072×10^{-5}	76	T_5	2.99438×10^{-5}
30	I_6	-2.41169×10^{-5}	75	I_2	2.95292×10^{-5}
29	I_5	-2.46252×10^{-5}	74	I_1	2.89847×10^{-5}
28	I_7	-2.46555×10^{-5}	73	I_5	2.88752×10^{-5}
27	T_2	-2.48211×10^{-5}	72	T_7	2.86388×10^{-5}
26	T_{11}	-2.55011×10^{-5}	71	E_2	2.84139×10^{-5}
25	G_1	-2.59324×10^{-5}	70	T_4	2.80539×10^{-5}
24	I_5	-2.65143×10^{-5}	69	I_3	2.74165×10^{-5}
23	I_3	-2.67472×10^{-5}	68	I_4	2.72663×10^{-5}
22	I_4	-2.70696×10^{-5}	67	I_5	2.65143×10^{-5}
21	T_5	-2.80576×10^{-5}	66	G_1	2.59324×10^{-5}
20	T_8	-2.83549×10^{-5}	65	T_{10}	2.54684×10^{-5}
19	E_2	-2.84139×10^{-5}	64	T_1	2.53643×10^{-5}
18	I_1	-2.85395×10^{-5}	63	I_5	2.46252×10^{-5}
17	I_5	-2.88752×10^{-5}	62	I_7	2.41863×10^{-5}
16	T_4	-2.93236×10^{-5}	61	I_6	2.40976×10^{-5}
15	I_2	-2.95129×10^{-5}	60	T_4	2.40287×10^{-5}
14	I_2	-3.00957×10^{-5}	59	I_1	2.37892×10^{-5}
13	T_6	-3.06290×10^{-5}	58	I_2	2.32486×10^{-5}
12	T_3	-3.09128×10^{-5}	57	T_2	2.31900×10^{-5}
11	T_{11}	-3.21644×10^{-5}	56	T_{11}	2.31065×10^{-5}
10	E_1	-3.26673×10^{-5}	55	A_2	2.27590×10^{-5}
9	I_6	-3.26778×10^{-5}	54	T_6	2.24773×10^{-5}
8	T_9	-3.31178×10^{-5}	53	T_{12}	2.24032×10^{-5}
7	I_1	-3.32404×10^{-5}	52	I_6	2.19877×10^{-5}
6	I_7	-3.37856×10^{-5}	51	T_9	2.09776×10^{-5}
5	I_4	-3.38387×10^{-5}	50	E_3	2.05540×10^{-5}
4	I_3	-3.44614×10^{-5}	49	I_3	2.04553×10^{-5}
3	A_3	-3.45590×10^{-5}	48	I_4	2.00964×10^{-5}
2	T_7	-3.47027×10^{-5}	47	I_7	1.90060×10^{-5}
1	T_1	-3.63805×10^{-5}	46	T_8	1.65836×10^{-5}

Table S15 Tunneling splitting pattern of the hexamer prism PR3 in the excited mode v_3.

State	Irrep	Energy level	State	Irrep	Energy level
45	T_7	-1.81838×10^{-6}	90	A_3	2.25690×10^{-5}
44	I_3	-2.92039×10^{-6}	89	T_1	2.24817×10^{-5}
43	I_4	-2.92536×10^{-6}	88	T_7	2.17947×10^{-5}
42	A_2	-3.42500×10^{-6}	87	I_7	2.13904×10^{-5}
41	T_{11}	-4.48132×10^{-6}	86	I_4	2.06496×10^{-5}
40	I_7	-4.79605×10^{-6}	85	I_3	2.04008×10^{-5}
39	T_4	-5.20888×10^{-6}	84	T_{11}	1.96441×10^{-5}
38	I_6	-6.07126×10^{-6}	83	I_6	1.95084×10^{-5}
37	I_2	-6.33894×10^{-6}	82	T_9	1.93543×10^{-5}
36	T_6	-6.90701×10^{-6}	81	I_1	1.89881×10^{-5}
35	T_9	-7.33363×10^{-6}	80	E_1	1.84768×10^{-5}
34	T_{10}	-7.46792×10^{-6}	79	T_8	1.66578×10^{-5}
33	I_1	-7.48717×10^{-6}	78	T_4	1.64538×10^{-5}
32	I_5	-8.15699×10^{-6}	77	I_2	1.61976×10^{-5}
31	T_1	-9.29463×10^{-6}	76	T_6	1.58826×10^{-5}
30	I_6	-9.38625×10^{-6}	75	T_5	1.46384×10^{-5}
29	G_1	-9.73965×10^{-6}	74	I_5	1.38445×10^{-5}
28	T_2	-1.01242×10^{-5}	73	I_3	1.36715×10^{-5}
27	I_5	-1.05027×10^{-5}	72	E_2	1.36690×10^{-5}
26	T_{12}	-1.06043×10^{-5}	71	I_4	1.36624×10^{-5}
25	T_6	-1.24006×10^{-5}	70	I_2	1.35205×10^{-5}
24	I_1	-1.24877×10^{-5}	69	I_1	1.32209×10^{-5}
23	I_3	-1.25666×10^{-5}	68	I_7	1.28185×10^{-5}
22	E_3	-1.28894×10^{-5}	67	T_3	1.22793×10^{-5}
21	I_4	-1.29081×10^{-5}	66	E_3	1.20434×10^{-5}
20	I_2	-1.35629×10^{-5}	65	T_2	1.08079×10^{-5}
19	E_2	-1.36690×10^{-5}	64	T_9	1.05483×10^{-5}
18	I_5	-1.38445×10^{-5}	63	I_5	1.05027×10^{-5}
17	I_7	-1.38942×10^{-5}	62	T_1	1.02279×10^{-5}
16	T_4	-1.46699×10^{-5}	61	G_1	9.73965×10^{-6}
15	T_3	-1.48412×10^{-5}	60	I_6	9.33382×10^{-6}
14	I_2	-1.53258×10^{-5}	59	I_5	8.15699×10^{-6}
13	T_5	-1.56833×10^{-5}	58	T_{12}	7.77373×10^{-6}
12	T_7	-1.65513×10^{-5}	57	I_1	7.67955×10^{-6}
11	E_1	-1.76308×10^{-5}	56	T_{11}	7.40619×10^{-6}
10	I_1	-1.82217×10^{-5}	55	T_3	6.83300×10^{-6}
9	T_{12}	-2.05844×10^{-5}	54	I_6	6.40930×10^{-6}
8	I_6	-2.06400×10^{-5}	53	I_2	6.35557×10^{-6}
7	T_{10}	-2.06899×10^{-5}	52	T_5	5.31593×10^{-6}
6	I_4	-2.15616×10^{-5}	51	I_7	5.22738×10^{-6}
5	I_3	-2.17213×10^{-5}	50	T_{10}	4.74282×10^{-6}
4	I_7	-2.24380×10^{-5}	49	A_1	4.27100×10^{-6}
3	T_8	-2.31697×10^{-5}	48	I_3	3.13588×10^{-6}
2	T_2	-2.32527×10^{-5}	47	I_4	3.08313×10^{-6}
1	A_4	-2.34150×10^{-5}	46	T_8	2.24098×10^{-6}

Table S16 Tunneling splitting pattern of the hexamer prism PR3 in the excited mode v_4.

State	Irrep	Energy level	State	Irrep	Energy level
45	A_3	-9.79100×10^{-6}	90	T_8	3.90240×10^{-5}
44	T_9	-1.30181×10^{-5}	89	I_7	3.81742×10^{-5}
43	T_{11}	-1.30615×10^{-5}	88	T_4	3.79011×10^{-5}
42	I_7	-1.31209×10^{-5}	87	I_3	3.77438×10^{-5}
41	I_6	-1.39634×10^{-5}	86	T_9	3.75985×10^{-5}
40	I_3	-1.49278×10^{-5}	85	I_5	3.67193×10^{-5}
39	I_4	-1.50013×10^{-5}	84	E_3	3.66817×10^{-5}
38	T_8	-1.54948×10^{-5}	83	I_6	3.66002×10^{-5}
37	I_7	-1.56462×10^{-5}	82	T_6	3.58141×10^{-5}
36	E_3	-1.60197×10^{-5}	81	I_1	3.54811×10^{-5}
35	T_1	-1.61217×10^{-5}	80	A_2	3.53870×10^{-5}
34	I_5	-1.65283×10^{-5}	79	T_{12}	3.53209×10^{-5}
33	T_7	-1.70833×10^{-5}	78	T_{10}	3.45923×10^{-5}
32	T_6	-1.88206×10^{-5}	77	I_4	3.44979×10^{-5}
31	T_{10}	-1.90530×10^{-5}	76	I_2	3.44800×10^{-5}
30	T_3	-1.94462×10^{-5}	75	T_2	3.41848×10^{-5}
29	I_2	-2.26130×10^{-5}	74	I_7	3.31287×10^{-5}
28	G_1	-2.31365×10^{-5}	73	E_2	3.30448×10^{-5}
27	I_6	-2.38644×10^{-5}	72	T_8	3.25198×10^{-5}
26	I_3	-2.41403×10^{-5}	71	T_1	3.21489×10^{-5}
25	I_1	-2.44355×10^{-5}	70	I_3	3.20101×10^{-5}
24	I_7	-2.57730×10^{-5}	69	I_4	3.04663×10^{-5}
23	I_5	-2.58465×10^{-5}	68	A_4	3.04530×10^{-5}
22	T_{11}	-2.64607×10^{-5}	67	I_6	2.98577×10^{-5}
21	T_{12}	-2.73012×10^{-5}	66	T_{11}	2.97312×10^{-5}
20	I_2	-2.74583×10^{-5}	65	I_5	2.58465×10^{-5}
19	T_4	-2.77232×10^{-5}	64	I_2	2.53181×10^{-5}
18	I_4	-2.84703×10^{-5}	63	T_4	2.52091×10^{-5}
17	I_6	-2.89286×10^{-5}	62	I_7	2.45612×10^{-5}
16	E_2	-3.30448×10^{-5}	61	G_1	2.31365×10^{-5}
15	T_2	-3.35888×10^{-5}	60	T_{12}	2.24333×10^{-5}
14	T_5	-3.37355×10^{-5}	59	I_1	2.16343×10^{-5}
13	T_9	-3.43714×10^{-5}	58	I_6	2.09605×10^{-5}
12	E_1	-3.44374×10^{-5}	57	T_5	1.93488×10^{-5}
11	I_1	-3.44394×10^{-5}	56	T_7	1.89806×10^{-5}
10	I_5	-3.67193×10^{-5}	55	T_6	1.83935×10^{-5}
9	T_7	-3.72843×10^{-5}	54	I_4	1.67341×10^{-5}
8	I_4	-3.82267×10^{-5}	53	I_5	1.65283×10^{-5}
7	T_5	-4.16623×10^{-5}	52	T_{10}	1.49137×10^{-5}
6	I_3	-4.24099×10^{-5}	51	I_2	1.38515×10^{-5}
5	I_2	-4.42403×10^{-5}	50	E_1	1.37754×10^{-5}
4	T_1	-4.64802×10^{-5}	49	I_3	1.17241×10^{-5}
3	T_3	-4.68537×10^{-5}	48	I_1	1.09704×10^{-5}
2	I_1	-5.05350×10^{-5}	47	T_3	1.02509×10^{-5}
1	A_1	-5.60490×10^{-5}	46	T_2	9.19506×10^{-6}

Table S17 Tunneling splitting pattern of the hexamer prism PR3 in the excited mode ν_5.

State	Irrep	Energy level	State	Irrep	Energy level
45	A_4	-2.80800×10^{-6}	90	T_7	4.23866×10^{-5}
44	I_7	-4.58610×10^{-6}	89	I_3	4.16068×10^{-5}
43	E_3	-5.11085×10^{-6}	88	I_7	4.10889×10^{-5}
42	T_{12}	-5.79107×10^{-6}	87	T_{10}	4.08461×10^{-5}
41	T_{10}	-5.83240×10^{-6}	86	T_3	4.07906×10^{-5}
40	I_7	-6.31888×10^{-6}	85	I_6	4.01358×10^{-5}
39	I_6	-6.55386×10^{-6}	84	E_3	4.01029×10^{-5}
38	T_8	-7.30454×10^{-6}	83	I_5	3.93158×10^{-5}
37	I_5	-7.32005×10^{-6}	82	T_9	3.93039×10^{-5}
36	T_9	-7.39807×10^{-6}	81	I_7	3.83832×10^{-5}
35	I_3	-8.09263×10^{-6}	80	T_{11}	3.78458×10^{-5}
34	T_4	-8.85105×10^{-6}	79	A_3	3.78000×10^{-5}
33	I_7	-2.38579×10^{-5}	78	T_2	2.86533×10^{-5}
32	I_6	-2.39606×10^{-5}	77	I_4	2.85638×10^{-5}
31	T_8	-2.40153×10^{-5}	76	T_{12}	2.77566×10^{-5}
30	E_2	-2.42978×10^{-5}	75	I_2	2.71673×10^{-5}
29	I_4	-2.44669×10^{-5}	74	I_6	2.67881×10^{-5}
28	T_{12}	-2.47735×10^{-5}	73	I_1	2.65241×10^{-5}
27	I_6	-2.54658×10^{-5}	72	T_1	2.64988×10^{-5}
26	T_{11}	-2.55733×10^{-5}	71	G_1	2.62210×10^{-5}
25	I_5	-2.55965×10^{-5}	70	I_3	2.62081×10^{-5}
24	T_6	-2.57059×10^{-5}	69	I_4	2.58900×10^{-5}
23	T_7	-2.58704×10^{-5}	68	I_5	2.55965×10^{-5}
22	I_2	-2.61003×10^{-5}	67	T_{11}	2.55275×10^{-5}
21	I_3	-2.62180×10^{-5}	66	T_6	2.55020×10^{-5}
20	G_1	-2.62210×10^{-5}	65	T_8	2.52999×10^{-5}
19	I_4	-2.67470×10^{-5}	64	I_7	2.52748×10^{-5}
18	I_4	-2.68439×10^{-5}	63	I_2	2.49519×10^{-5}
17	T_2	-2.70410×10^{-5}	62	T_5	2.48520×10^{-5}
16	I_2	-2.74500×10^{-5}	61	T_7	2.44958×10^{-5}
15	T_5	-2.75807×10^{-5}	60	E_2	2.42978×10^{-5}
14	I_1	-2.79486×10^{-5}	59	I_6	2.40483×10^{-5}
13	T_1	-2.80227×10^{-5}	58	I_4	2.36040×10^{-5}
12	T_{10}	-3.78217×10^{-5}	57	T_5	8.74873×10^{-6}
11	I_3	-3.86306×10^{-5}	56	I_1	8.22472×10^{-6}
10	I_5	-3.93158×10^{-5}	55	E_1	7.94878×10^{-6}
9	T_2	-3.94122×10^{-5}	54	I_5	7.32005×10^{-6}
8	T_4	-3.94268×10^{-5}	53	T_4	7.26583×10^{-6}
7	I_2	-4.01201×10^{-5}	52	I_2	6.55919×10^{-6}
6	I_1	-4.04976×10^{-5}	51	A_1	6.02000×10^{-6}
5	T_3	-4.07182×10^{-5}	50	T_3	5.94767×10^{-6}
4	T_6	-4.08081×10^{-5}	49	T_9	5.89419×10^{-6}
3	A_2	-4.10120×10^{-5}	48	I_1	5.63851×10^{-6}
2	I_1	-4.19251×10^{-5}	47	I_3	5.12639×10^{-6}
1	E_1	-4.29408×10^{-5}	46	T_1	4.33196×10^{-6}

Table S18 Tunneling splitting pattern of the hexamer prism PR3 in the excited mode ν_6.

State	Irrep	Energy level	State	Irrep	Energy level
45	T_1	-1.36763×10^{-4}	90	A_2	1.81600×10^{-4}
44	A_3	-1.39000×10^{-4}	89	I_1	1.77789×10^{-4}
43	I_4	-1.39501×10^{-4}	88	T_8	1.76033×10^{-4}
42	I_3	-1.40218×10^{-4}	87	T_6	1.75279×10^{-4}
41	T_8	-1.41602×10^{-4}	86	T_2	1.74337×10^{-4}
40	T_9	-1.42541×10^{-4}	85	I_2	1.73532×10^{-4}
39	T_{11}	-1.42998×10^{-4}	84	I_4	1.72896×10^{-4}
38	I_6	-1.43288×10^{-4}	83	T_4	1.72866×10^{-4}
37	T_6	-1.43990×10^{-4}	82	I_7	1.71528×10^{-4}
36	I_7	-1.44732×10^{-4}	81	I_3	1.70084×10^{-4}
35	I_1	-1.45468×10^{-4}	80	I_5	1.69442×10^{-4}
34	I_2	-1.46278×10^{-4}	79	T_9	1.68570×10^{-4}
33	I_7	-1.47465×10^{-4}	78	E_2	1.68111×10^{-4}
32	I_5	-1.47897×10^{-4}	77	E_3	1.68089×10^{-4}
31	T_7	-1.49081×10^{-4}	76	I_6	1.67274×10^{-4}
30	T_4	-1.49188×10^{-4}	75	I_3	1.67080×10^{-4}
29	T_3	-1.50787×10^{-4}	74	T_{12}	1.66710×10^{-4}
28	G_1	-1.51922×10^{-4}	73	T_1	1.64271×10^{-4}
27	E_3	-1.52689×10^{-4}	72	T_{10}	1.63816×10^{-4}
26	E_1	-1.53002×10^{-4}	71	I_6	1.62735×10^{-4}
25	I_5	-1.54378×10^{-4}	70	T_{11}	1.62596×10^{-4}
24	T_{10}	-1.54935×10^{-4}	69	T_8	1.62570×10^{-4}
23	I_2	-1.56081×10^{-4}	68	I_4	1.61944×10^{-4}
22	I_3	-1.56329×10^{-4}	67	I_7	1.59528×10^{-4}
21	I_6	-1.57623×10^{-4}	66	T_4	1.57922×10^{-4}
20	I_1	-1.58423×10^{-4}	65	I_2	1.55078×10^{-4}
19	T_{11}	-1.58598×10^{-4}	64	A_4	1.54400×10^{-4}
18	T_5	-1.60229×10^{-4}	63	I_5	1.54378×10^{-4}
17	I_4	-1.60704×10^{-4}	62	I_7	1.53300×10^{-4}
16	T_{12}	-1.61075×10^{-4}	61	G_1	1.51922×10^{-4}
15	I_7	-1.61360×10^{-4}	60	I_1	1.50713×10^{-4}
14	I_6	-1.61796×10^{-4}	59	T_6	1.50311×10^{-4}
13	T_2	-1.64504×10^{-4}	58	T_{12}	1.48765×10^{-4}
12	T_9	-1.65029×10^{-4}	57	I_6	1.48099×10^{-4}
11	E_2	-1.68111×10^{-4}	56	I_5	1.47897×10^{-4}
10	I_5	-1.69442×10^{-4}	55	T_{10}	1.45519×10^{-4}
9	I_4	-1.72833×10^{-4}	54	T_5	1.41149×10^{-4}
8	T_7	-1.73271×10^{-4}	53	T_7	1.40751×10^{-4}
7	I_3	-1.76326×10^{-4}	52	I_4	1.38198×10^{-4}
6	T_5	-1.77920×10^{-4}	51	I_2	1.38164×10^{-4}
5	I_2	-1.79816×10^{-4}	50	E_1	1.37602×10^{-4}
4	T_1	-1.81908×10^{-4}	49	T_3	1.35786×10^{-4}
3	T_3	-1.81999×10^{-4}	48	I_3	1.35709×10^{-4}
2	I_1	-1.88595×10^{-4}	47	I_1	1.33184×10^{-4}
1	A_1	-1.97000×10^{-4}	46	T_2	1.29167×10^{-4}

Table S19 Tunneling splitting pattern of the hexamer prism PR3 in the excited mode ν_7.

State	Irrep	Energy level	State	Irrep	Energy level
45	E_3	-1.81503×10^{-5}	90	A_4	3.64400×10^{-5}
44	I_7	-1.84741×10^{-5}	89	T_{10}	3.63277×10^{-5}
43	T_{11}	-1.87606×10^{-5}	88	I_7	3.62320×10^{-5}
42	I_6	-1.97960×10^{-5}	87	T_8	3.60250×10^{-5}
41	T_{10}	-2.01565×10^{-5}	86	I_3	3.58972×10^{-5}
40	I_5	-2.05403×10^{-5}	85	I_6	3.57291×10^{-5}
39	I_7	-2.07412×10^{-5}	84	T_4	3.56968×10^{-5}
38	T_9	-2.12057×10^{-5}	83	I_5	3.55895×10^{-5}
37	T_8	-2.12610×10^{-5}	82	T_{12}	3.54980×10^{-5}
36	T_7	-2.14980×10^{-5}	81	E_2	3.44646×10^{-5}
35	I_3	-2.16475×10^{-5}	80	I_4	3.43580×10^{-5}
34	I_4	-2.17271×10^{-5}	79	I_2	3.39986×10^{-5}
33	T_3	-2.23694×10^{-5}	78	T_2	3.39330×10^{-5}
32	T_6	-2.30984×10^{-5}	77	T_6	3.38848×10^{-5}
31	T_1	-2.39186×10^{-5}	76	I_1	3.32253×10^{-5}
30	A_3	-2.40400×10^{-5}	75	A_2	3.17600×10^{-5}
29	I_6	-2.44277×10^{-5}	74	T_9	3.10192×10^{-5}
28	G_1	-2.47530×10^{-5}	73	I_7	3.09128×10^{-5}
27	I_3	-2.47649×10^{-5}	72	E_3	3.05503×10^{-5}
26	I_2	-2.50404×10^{-5}	71	T_8	2.93960×10^{-5}
25	T_{12}	-2.59776×10^{-5}	70	I_6	2.85343×10^{-5}
24	I_1	-2.64316×10^{-5}	69	I_3	2.83975×10^{-5}
23	I_5	-2.69206×10^{-5}	68	I_5	2.69206×10^{-5}
22	I_4	-2.72679×10^{-5}	67	T_{12}	2.69196×10^{-5}
21	I_2	-2.75661×10^{-5}	66	T_1	2.68649×10^{-5}
20	T_2	-2.83786×10^{-5}	65	I_4	2.66678×10^{-5}
19	T_4	-2.83956×10^{-5}	64	T_{11}	2.62500×10^{-5}
18	I_7	-2.91171×10^{-5}	63	I_7	2.59877×10^{-5}
17	T_5	-3.00526×10^{-5}	62	I_2	2.50954×10^{-5}
16	I_1	-3.03528×10^{-5}	61	G_1	2.47530×10^{-5}
15	E_1	-3.06296×10^{-5}	60	T_4	2.44588×10^{-5}
14	T_{11}	-3.15295×10^{-5}	59	I_6	2.43421×10^{-5}
13	I_6	-3.19818×10^{-5}	58	T_5	2.39835×10^{-5}
12	T_9	-3.38535×10^{-5}	57	T_7	2.36673×10^{-5}
11	T_7	-3.39293×10^{-5}	56	I_4	2.34767×10^{-5}
10	E_2	-3.44646×10^{-5}	55	I_1	2.24650×10^{-5}
9	I_4	-3.55074×10^{-5}	54	T_6	2.09737×10^{-5}
8	I_5	-3.55895×10^{-5}	53	I_5	2.05403×10^{-5}
7	I_3	-3.70690×10^{-5}	52	T_{10}	2.02688×10^{-5}
6	T_5	-3.80910×10^{-5}	51	I_2	1.97105×10^{-5}
5	I_2	-3.85980×10^{-5}	50	I_3	1.91867×10^{-5}
4	T_1	-3.93863×10^{-5}	49	T_2	1.84856×10^{-5}
3	T_3	-3.94810×10^{-5}	48	E_1	1.82296×10^{-5}
2	I_1	-4.15669×10^{-5}	47	I_1	1.78609×10^{-5}
1	A_1	-4.41600×10^{-5}	46	T_3	1.76904×10^{-5}

Table S20 Tunneling splitting pattern of the hexamer prism PR3 in the excited mode ν_8.

State	Irrep	Energy level	State	Irrep	Energy level
45	I_4	-5.33610×10^{-7}	90	A_2	4.44200×10^{-5}
44	I_3	-7.90425×10^{-7}	89	T_8	4.12185×10^{-5}
43	I_7	-2.89017×10^{-6}	88	I_1	3.95196×10^{-5}
42	T_9	-3.28502×10^{-6}	87	T_1	3.85963×10^{-5}
41	I_1	-3.44220×10^{-6}	86	I_3	3.81494×10^{-5}
40	T_6	-3.55671×10^{-6}	85	I_4	3.81381×10^{-5}
39	I_6	-3.98009×10^{-6}	84	T_2	3.77719×10^{-5}
38	I_2	-4.31821×10^{-6}	83	I_7	3.70031×10^{-5}
37	T_{11}	-4.52250×10^{-6}	82	E_3	3.55463×10^{-5}
36	T_4	-4.96492×10^{-6}	81	T_6	3.51126×10^{-5}
35	T_{11}	-1.01853×10^{-5}	80	I_2	3.50290×10^{-5}
34	I_5	-1.06894×10^{-5}	79	T_4	3.49445×10^{-5}
33	I_6	-1.09062×10^{-5}	78	T_9	3.35109×10^{-5}
32	G_1	-1.12752×10^{-5}	77	I_6	3.34200×10^{-5}
31	T_9	-1.16059×10^{-5}	76	T_{11}	3.33278×10^{-5}
30	I_5	-1.16666×10^{-5}	75	I_1	2.91121×10^{-5}
29	T_3	-1.28689×10^{-5}	74	I_3	2.80764×10^{-5}
28	I_2	-1.36790×10^{-5}	73	I_4	2.80218×10^{-5}
27	E_2	-1.37233×10^{-5}	72	T_8	2.76144×10^{-5}
26	I_5	-1.38643×10^{-5}	71	E_1	2.67517×10^{-5}
25	T_5	-1.44445×10^{-5}	70	I_7	2.61732×10^{-5}
24	A_4	-1.70200×10^{-5}	69	T_7	2.43418×10^{-5}
23	T_2	-1.79683×10^{-5}	68	A_3	1.86200×10^{-5}
22	T_8	-2.28129×10^{-5}	67	T_1	1.77391×10^{-5}
21	I_7	-2.47122×10^{-5}	66	T_4	1.44405×10^{-5}
20	I_3	-2.77423×10^{-5}	65	I_5	1.38643×10^{-5}
19	I_4	-2.78345×10^{-5}	64	E_2	1.37233×10^{-5}
18	T_7	-2.79189×10^{-5}	63	I_2	1.36747×10^{-5}
17	E_1	-2.83517×10^{-5}	62	T_6	1.28642×10^{-5}
16	I_1	-3.05445×10^{-5}	61	I_5	1.16666×10^{-5}
15	T_{10}	-3.19115×10^{-5}	60	T_{12}	1.16111×10^{-5}
14	I_6	-3.20111×10^{-5}	59	G_1	1.12752×10^{-5}
13	T_{12}	-3.21091×10^{-5}	58	I_6	1.09141×10^{-5}
12	E_3	-3.39463×10^{-5}	57	I_5	1.06894×10^{-5}
11	I_7	-3.54633×10^{-5}	56	T_{10}	1.01981×10^{-5}
10	T_5	-3.63861×10^{-5}	55	T_5	4.81056×10^{-6}
9	I_2	-3.64647×10^{-5}	54	T_{10}	4.69341×10^{-6}
8	T_3	-3.65425×10^{-5}	53	I_6	4.16331×10^{-6}
7	T_2	-3.84236×10^{-5}	52	I_2	4.15827×10^{-6}
6	I_4	-3.84603×10^{-5}	51	T_{12}	3.47804×10^{-6}
5	I_3	-3.84695×10^{-5}	50	T_3	3.39147×10^{-6}
4	T_1	-3.93154×10^{-5}	49	I_1	3.19181×10^{-6}
3	T_7	-4.08429×10^{-5}	48	I_7	3.08938×10^{-6}
2	I_1	-4.10369×10^{-5}	47	I_3	7.76532×10^{-7}
1	A_1	-4.60200×10^{-5}	46	I_4	6.68479×10^{-7}

Table S21 Tunneling splitting pattern of the hexamer prism PR3 in the excited mode ν_9.

State	Irrep	Energy level	State	Irrep	Energy level
45	T_7	-7.42568×10^{-6}	90	A_4	5.06000×10^{-5}
44	A_1	-9.80000×10^{-6}	89	T_2	4.72127×10^{-5}
43	I_7	-1.50773×10^{-5}	88	I_7	4.57004×10^{-5}
42	I_3	-1.56942×10^{-5}	87	I_3	4.37328×10^{-5}
41	I_4	-1.58154×10^{-5}	86	I_4	4.36243×10^{-5}
40	I_1	-1.71520×10^{-5}	85	T_8	4.34756×10^{-5}
39	T_{10}	-1.79368×10^{-5}	84	T_7	4.17734×10^{-5}
38	I_6	-1.89392×10^{-5}	83	T_{10}	4.17379×10^{-5}
37	T_5	-1.95511×10^{-5}	82	I_1	4.16651×10^{-5}
36	T_{12}	-1.99718×10^{-5}	81	I_6	4.11782×10^{-5}
35	I_2	-2.05183×10^{-5}	80	T_{12}	4.05743×10^{-5}
34	T_3	-2.15089×10^{-5}	79	E_1	3.92716×10^{-5}
33	E_3	-2.35645×10^{-5}	78	T_5	3.89626×10^{-5}
32	T_2	-2.50817×10^{-5}	77	I_2	3.83747×10^{-5}
31	T_1	-2.58084×10^{-5}	76	T_3	3.77334×10^{-5}
30	T_3	-2.60245×10^{-5}	75	I_7	3.29760×10^{-5}
29	I_5	-2.63669×10^{-5}	74	I_4	3.17579×10^{-5}
28	T_{11}	-2.67979×10^{-5}	73	I_3	3.15845×10^{-5}
27	I_2	-2.76416×10^{-5}	72	T_{12}	2.99975×10^{-5}
26	G_1	-2.78280×10^{-5}	71	I_1	2.98718×10^{-5}
25	I_5	-2.80225×10^{-5}	70	I_5	2.96406×10^{-5}
24	I_6	-2.84224×10^{-5}	69	T_4	2.92104×10^{-5}
23	E_2	-2.84253×10^{-5}	68	E_2	2.84253×10^{-5}
22	T_5	-2.92115×10^{-5}	67	I_6	2.84237×10^{-5}
21	I_5	-2.96406×10^{-5}	66	I_5	2.80225×10^{-5}
20	T_9	-2.99957×10^{-5}	65	G_1	2.78280×10^{-5}
19	I_7	-3.08950×10^{-5}	64	I_2	2.76396×10^{-5}
18	I_3	-3.16983×10^{-5}	63	E_3	2.71645×10^{-5}
17	I_4	-3.17229×10^{-5}	62	T_{10}	2.67989×10^{-5}
16	I_1	-3.19431×10^{-5}	61	I_5	2.63669×10^{-5}
15	T_9	-3.82723×10^{-5}	60	T_6	2.60196×10^{-5}
14	I_6	-3.89036×10^{-5}	59	T_2	2.48691×10^{-5}
13	T_{11}	-3.94848×10^{-5}	58	T_1	2.36143×10^{-5}
12	T_6	-4.00207×10^{-5}	57	T_9	2.12680×10^{-5}
11	T_7	-4.05477×10^{-5}	56	I_6	2.02632×10^{-5}
10	I_2	-4.06336×10^{-5}	55	T_6	2.02011×10^{-5}
9	T_4	-4.11994×10^{-5}	54	T_{11}	1.92827×10^{-5}
8	T_8	-4.22898×10^{-5}	53	I_2	1.91792×10^{-5}
7	I_7	-4.26843×10^{-5}	52	T_4	1.81890×10^{-5}
6	E_1	-4.28716×10^{-5}	51	I_7	1.71803×10^{-5}
5	I_4	-4.35915×10^{-5}	50	I_3	1.57865×10^{-5}
4	I_3	-4.37113×10^{-5}	49	I_4	1.57477×10^{-5}
3	I_1	-4.46998×10^{-5}	48	I_1	1.50581×10^{-5}
2	A_3	-4.70000×10^{-5}	47	T_8	8.61421×10^{-6}
1	T_1	-4.84059×10^{-5}	46	A_2	6.20000×10^{-6}

Table S22 Ground-state tunneling matrix elements for various relevant degenerate rearrangements of the water hexamer prisms PR2 and PR3 (see main article for details) on MB-pol and WHBB PES in cm^{-1}. The number of MAP discretization points in calculations using WHBB PES was set to $N = 100$.

	MB-Pol	WHBB
$h^{\mathrm{PR2}}_{\mathrm{AD}}/10^{-7}$	-8.91	-12.1
$h^{\mathrm{PR2}}_{\tilde{\mathrm{A}}\mathrm{D}}/10^{-8}$	-5.81	-5.59
$h^{\mathrm{PR2}}_{\mathrm{A}\tilde{\mathrm{D}}}/10^{-7}$	-8.97	-19.7
$h^{\mathrm{PR2}}_{\mathrm{rotB}}/10^{-5}$	-0.700	-0.575
$h^{\mathrm{PR2}}_{\mathrm{rotD}}/10^{-7}$	-8.11	-7.94
$h^{\mathrm{PR2}}_{\mathrm{rotF}}/10^{-6}$	-0.859	-0.667
$h^{\mathrm{PR3}}_{\mathrm{AD}}/10^{-5}$	-2.42	-2.66
$h^{\mathrm{PR3}}_{\tilde{\mathrm{A}}\mathrm{D}}/10^{-7}$	-8.81	-13.6
$h^{\mathrm{PR3}}_{\mathrm{BE}}/10^{-6}$	-2.90	-1.82
$h^{\mathrm{PR3}}_{\tilde{\mathrm{B}}\mathrm{E}}/10^{-6}$	-5.30	-3.08
$h^{\mathrm{PR3}}_{\mathrm{rotB}}/10^{-6}$	-1.01	-0.768
$h^{\mathrm{PR3}}_{\mathrm{rotE}}/10^{-7}$	-5.36	-5.23
$h^{\mathrm{PR3}}_{\mathrm{rotF}}/10^{-7}$	-2.51	-2.43
